# Comparison of Combined Parenteral and Oral Hormonal Contraceptives: A Systematic Review and Meta-Analysis of Randomized Trials

**DOI:** 10.3390/jcm13020575

**Published:** 2024-01-19

**Authors:** Gábor Vleskó, Fanni Adél Meznerics, Péter Hegyi, Brigitta Teutsch, Márkó Unicsovics, Zoltán Sipos, Péter Fehérvári, Nándor Ács, Szabolcs Várbíró, Márton Keszthelyi

**Affiliations:** 1 Department of Obstetrics and Gynecology, Semmelweis University, 1082 Budapest, Hungary; vlesko.gabor@semmelweis.hu (G.V.); unicsovics.marko@semmelweis.hu (M.U.); acs.nandor@semmelweis.hu (N.Á.); keszthelyi.marton@semmelweis.hu (M.K.); 2 Centre for Translational Medicine, Semmelweis University, 1085 Budapest, Hungary; meznerics.fanni.adel@semmelweis.hu (F.A.M.); hegyi.peter@semmelweis.hu (P.H.); teutsch.brigitta@semmelweis.hu (B.T.); fehervari.peter@univet.hu (P.F.); 3 Department of Dermatology, Venereology and Dermatooncology, Faculty of Medicine, Semmelweis University, 1082 Budapest, Hungary; 4 Institute for Translational Medicine, Medical School, University of Pécs, 7621 Pécs, Hungary; sizfab.t.jpte@pte.hu; 5 Institute of Pancreatic Diseases, Semmelweis University, 1085 Budapest, Hungary; 6 Institute of Bioanalysis, Medical School, University of Pécs, 7621 Pécs, Hungary; 7 Department of Biostatistics, University of Veterinary Medicine, 1078 Budapest, Hungary; 8 Workgroup of Research Management, Doctoral School, Semmelweis University, 1085 Budapest, Hungary

**Keywords:** hormonal contraception, vaginal ring, contraceptive patch, combined parenteral contraception, combined oral contraception

## Abstract

Background: Delivering contraceptive hormones through a transdermal patch or a vaginal ring might have advantages over the traditional oral route. Objectives: To compare the effectiveness, compliance, and side effect profile of oral and parenteral drug administration methods. Methods: We performed a systematic literature search in four medical databases—MEDLINE (via PubMed), Cochrane Library (CENTRAL), Embase, and Scopus—from inception to 20 November 2022. Randomized controlled trials assessing the efficacy, compliance, and adverse event profile of combined parenteral and oral hormonal contraceptives were included. Results: Our systematic search provided 3952 records; after duplicate removal, we screened 2707 duplicate-free records. A total of 13 eligible studies were identified after title, abstract, and full-text selection. We observed no significant difference in contraceptive efficacy (Pearl Index) between oral and parenteral drug administration (MD = −0.06, CI: −0.66–0.53; I^2^ = 0%). We found significant subgroup differences between parenteral methods in terms of compliance (χ^2^ = 4.32, *p* =0.038, I^2^ = 80%) and certain adverse events: breast discomfort (χ^2^ = 19.04, *p* =0.001, I^2^ = 80%), nausea (χ^2^ = 8.04, *p* =0.005, I^2^ = 75%), and vomiting (χ^2^ = 9.30, *p* =0.002; I^2^ = 72%). Conclusion: Both parenteral and oral contraceptives can be used as an effective contraceptive method, and the route of administration should be tailored to patient needs and adverse event occurrence.

## 1. Introduction

Hormonal contraception has been widely used for decades and has constantly evolved since it was first introduced [1]. The efficacy of hormonal contraceptives depends largely on patient compliance [2,3,4].

Alternative methods of administration have been developed to reduce the side effect profile while decreasing hormonal levels [5,6,7,8]. Transdermal and vaginal administration allows lower hormonal dosage requirements due to increased bioavailability and provides a convenient and discrete option for women who prefer non-oral routes of administration [9,10]. Nevertheless, the combined oral contraceptive (COC) pill remains the most commonly used hormonal contraceptive method.

Contraceptive pills need to be taken consistently and at the same time every day to be effective. Missing pills or taking them at different times can reduce effectiveness [11]. For women who struggle with daily pill intake, transdermal patches or vaginal rings might be good alternatives [12].

While the existing literature has extensively explored the safety and efficacy of combined parenteral contraception, a critical observation reveals a noticeable temporal gap [13]. Despite the wealth of studies on both contraceptive modalities, a comprehensive synthesis of the latest evidence is conspicuously absent in the literature. This underscores the need for an updated meta-analysis to offer insights into the evolving landscape of combined parenteral contraception in comparison to combined oral contraception.

The aim of our research was to review and compare the efficacy, compliance, and safety of the combined parenteral contraceptive methods with the COC pill. Reducing the side effect profile and increasing compliance is a crucial public health concern due to its medical and financial implications. The outcomes of our analysis are poised to fill the existing temporal void in the literature, providing clinicians, researchers, and policymakers with timely and relevant insights that can inform contraceptive decision making and guide future research directions in this dynamic field [14].

## 2. Materials and Methods

Our systematic review and meta-analysis followed the PRISMA (Preferred Reporting Items for Systematic Reviews and Meta-Analyses) 2020 Statement [15]. The review protocol was registered on PROSPERO according to the recommendations of the Cochrane Prognosis Methods Group. The protocol of the study was registered on PROSPERO under registration number CRD42022374644 (see https://www.crd.york.ac.uk/prospero/, accessed on 20 November 2022). Respecting the protocol, we also searched the Scopus database.

A systematic literature search was performed in four medical databases—MEDLINE (via PubMed), Cochrane Library (CENTRAL), Embase, and Scopus—from inception to 20 November 2022 using the following search key: “(contraception or contraceptive) and (ring or patch or transdermal) and random*”. Manual searches involved a meticulous review of reference lists of relevant articles and key journals to identify additional studies that may not have been captured through electronic database searches alone. This approach was undertaken to thoroughly explore the existing literature and minimize the risk of overlooking relevant randomized controlled trials (RCTs) that might contribute valuable data to our meta-analysis.

The following population–intervention–control–outcome (PICO) framework was used:

P—Women of reproductive age;
I—Combined parenteral contraceptives (transdermal patch, vaginal ring);
C—Combined oral contraceptives (COCs);
O—Primary outcome: Pearl Index, secondary outcomes: compliance, adverse events.

The following inclusion and exclusion criteria were applied:

### 2.1. Inclusion Criteria

Studies were limited to randomized controlled trials (RCTs) to ensure a high level of methodological rigor. Only studies published in peer-reviewed journals were considered. The participants in the selected studies were women of fertile age. The intervention involved combined parenteral contraception (transdermal patches and vaginal rings). Studies reporting outcomes related to efficacy, adverse events, and cycle control were included. Reports had to identify the specific contraceptive methods used.

### 2.2. Exclusion Criteria

Non-randomized study designs were excluded. Studies published in non-peer-reviewed sources or grey literature were not considered. Studies with insufficient data or unclear methodology were excluded. We excluded studies focused on women with specific health problems, such as HIV or autoimmune diseases. We also excluded studies of contraceptives as a treatment for specific disorders such as abnormal uterine bleeding, acne, hirsutism, or polycystic ovary syndrome.

We used EndNote 20 (Clarivate Analytics, Philadelphia, PA, USA) to select the retrieved articles. After removing duplicates, two independent authors screened the library separately by title and abstract, then by full text (G.V., M.U.). Cohen’s kappa coefficient (κ) was calculated after each selection process to measure interrater reliability. Disagreements were resolved after each step by a third author (M.K.).

The odds ratio with 95% CI was applied to the effect measures of all outcomes. To calculate the odds ratio, the total number of patients in each group and those with the event of interest were extracted from each study. Raw data from the selected studies were pooled using a random effect model with the Mantel–Haenszel method and the Hartung–Knapp adjustment [16,17,18,19,20].

We used the Paule–Mandel method to estimate τ^2^ and the Q profile method for calculating the confidence interval of τ^2^. To evaluate publication bias, we used a funnel plot of the logarithm of the effect size and a comparison with the standard error for each trial.

Statistical heterogeneity across trials was assessed by means of the Cochrane Q test, and the I^2^ statistic values [21].

Outlier and influence analyses were carried out following the recommendations of Harrer et al. and Viechtbauer and Cheung [17,22].

We used forest plots to graphically summarize results. Where applicable, we reported the prediction intervals (i.e., the expected range of effects of future studies) of results following the recommendations of IntHout et al. [23].

All analyses were carried out using the R 4.1.3 (R Core Team 2021), using the packages ‘meta’ and ‘diameter’ [24].

The efficacy was assessed using the Pearl Index. The Pearl Index, also known as the Pearl Rate or Pearl Control Index, is a measure used to indicate the effectiveness of a contraceptive method in preventing pregnancies. The Pearl Index is defined as the number of pregnancies per 100 woman-years using a specific contraceptive method. In other words, it represents the number of unintended pregnancies that happen while using a contraceptive method over a year of usage for 100 women. The lower the Pearl Index, the more effective the contraceptive method is in preventing pregnancy. Compliance was measured using self-reported patient diaries; one study assessed compliance by measuring plasma steroid levels in addition to diaries [25].

The quality assessment of the outcomes was performed separately by two reviewers (M.U, G.V.) using the RoB 2 tool for assessing the risk of bias [26]. Disagreements were resolved by a third person (F.M.).

To assess the quality of the evidence, we followed the recommendation of the “Grades of Recommendation, Assessment, Development, and Evaluation (GRADE)” workgroup [27].

## 3. Results

### 3.1. Search and Selection, Characteristics of the Studies Included

**Figure 1:**
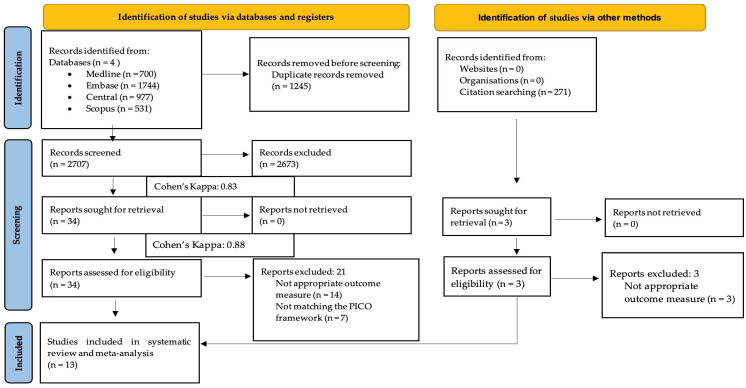
PRISMA flow diagram of the screening and selection process.

Our systematic search resulted in 3952 records; after duplicate removal, we reviewed 2707 duplicate-free records. A total of 13 eligible studies were identified after title, abstract, and full-text selection [25,28,29,30,31,32,33,34,35,36,37,38,39]. A summary of the selection process is shown in Figure 1.

The characteristics of the studies identified for the systematic review and meta-analysis, as well as the patient characteristics of the studies included, are detailed in Table 1.

### 3.2. Pearl Index for the Assessment of Contraceptive Efficacy

**Figure 2:**
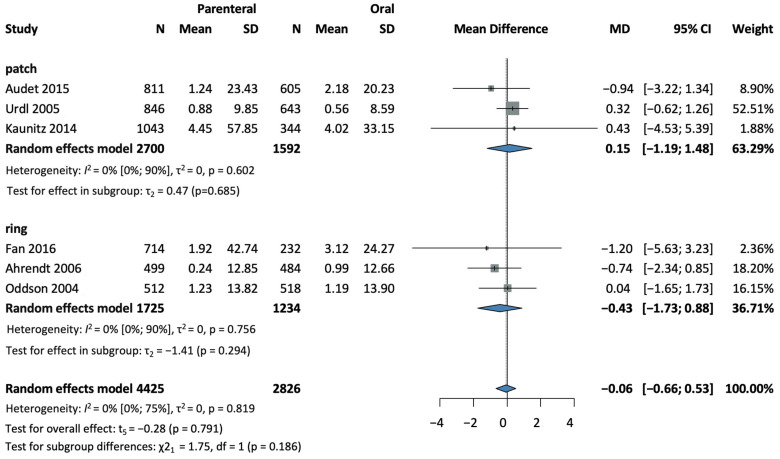
Forest plot of efficacy comparing combined parenteral and oral hormonal contraceptives [28,30,32,35,37,38].

Six studies [28,30,32,35,37,38], covering a total of 7251 patients, were selected for analysis. No difference in contraceptive efficacy was found between the parenteral and the oral administration groups (MD = −0.06, CI: −0.66–0.53; I^2^ = 0%). In a subgroup analysis according to the method of administration, three studies [28,32,37], with 2959 patients in the patch group, (MD = 0.15, CI: −1.19–1.48; I^2^ = 0%) and three studies [30,35,38], with 4292 patients in the ring group (MD = −0.43, CI: −1.73–0.88; I^2^ = 0%) found similar results (see Figure 2). No statistically significant difference was detected (χ^2^ = 1.75, df = 1, *p* = 0.186) between the transdermal patch and the vaginal ring subgroups (see Figure 2).

### 3.3. Compliance

**Figure 3:**
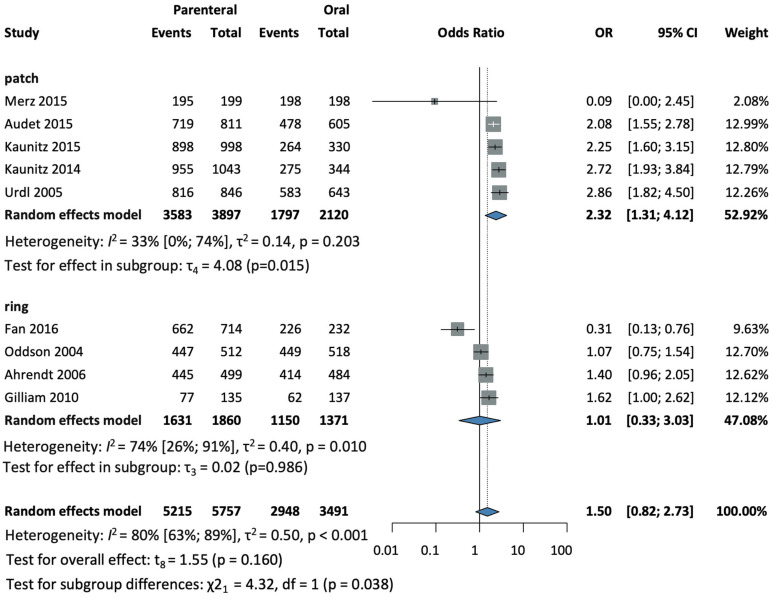
Forest plot of compliance with combined parenteral and oral hormonal contraceptives [25,28,30,31,32,34,35,37,38].

Nine studies [25,28,30,31,32,34,35,37,38], with a total of 9248 patients, were selected for analysis. The parenteral group had increased odds of better compliance (OR = 1.5, CI: 0.82–2.73; I^2^ = 80%) (see Figure 3).

In a subgroup analysis according to the method of administration, a total of five studies [25,28,31,32,37], with a total of 6017 patients in the patch subgroup, were selected for analysis. Significantly better compliance was measured in patients using the patch than in the oral group with an increased odds ratio (OR = 2.32, CI: 1.31–4.12; I^2^ = 33%) (see Figure 3).

In the ring subgroup, a total of four studies [30,34,35,38], involving a total of 3231 patients, found no difference compared to the oral group (OR = 1.01, CI: 0.33–3.03; I^2^ = 74% (see Figure 3).

The test for subgroup differences suggests that there was a statistically significant subgroup difference between the patch and ring subgroups, with the transdermal patch having significantly better compliance (χ^2^ = 4.32, df = 1, *p* = 0.038).

### 3.4. Adverse Events

#### 3.4.1. Breast Discomfort

**Figure 4:**
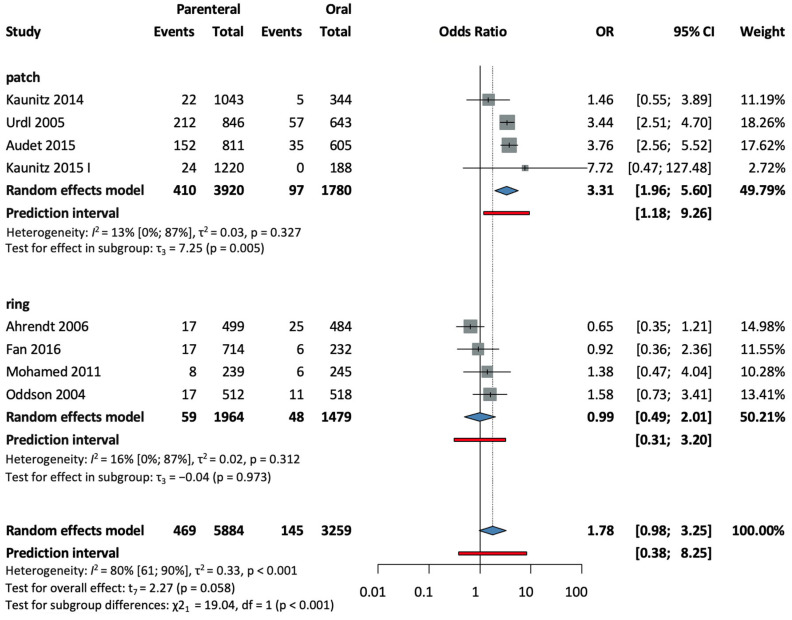
Forest plot of breast discomfort, comparing combined parenteral and oral hormonal contraceptives [28,30,32,33,35,37,38,39].

Eight studies [28,30,32,33,35,37,38,39], with a total of 9143 patients, were selected for analysis. The parenteral group was found to have clinically relevant increased odds for developing breast discomfort (OR = 1.78, CI: 0.98–3.25; I^2^ = 80%) (see Figure 4).

In a subgroup analysis according to the method of administration, four studies [28,32,33,37] were selected for analysis in the patch subgroup, with a total of 5700 patients. A statistically significant increase in breast discomfort was measured compared to the oral group (*p* = 0.05, OR = 3.31, CI: 1.96–5.60; I^2^ = 13%) (see Figure 4). Four studies [30,35,38,39] in the ring subgroup, with a total of 3443 patients, found no significant difference versus the control (OR = 0.99, CI: 0.49–2.01; I^2^ = 16%) (see Figure 4).

The test for subgroup differences suggests that there was a statistically significant subgroup difference (χ^2^ = 19.04, df = 1, *p* = 0.001).

#### 3.4.2. Vomiting and Nausea

##### Vomiting

**Figure 5:**
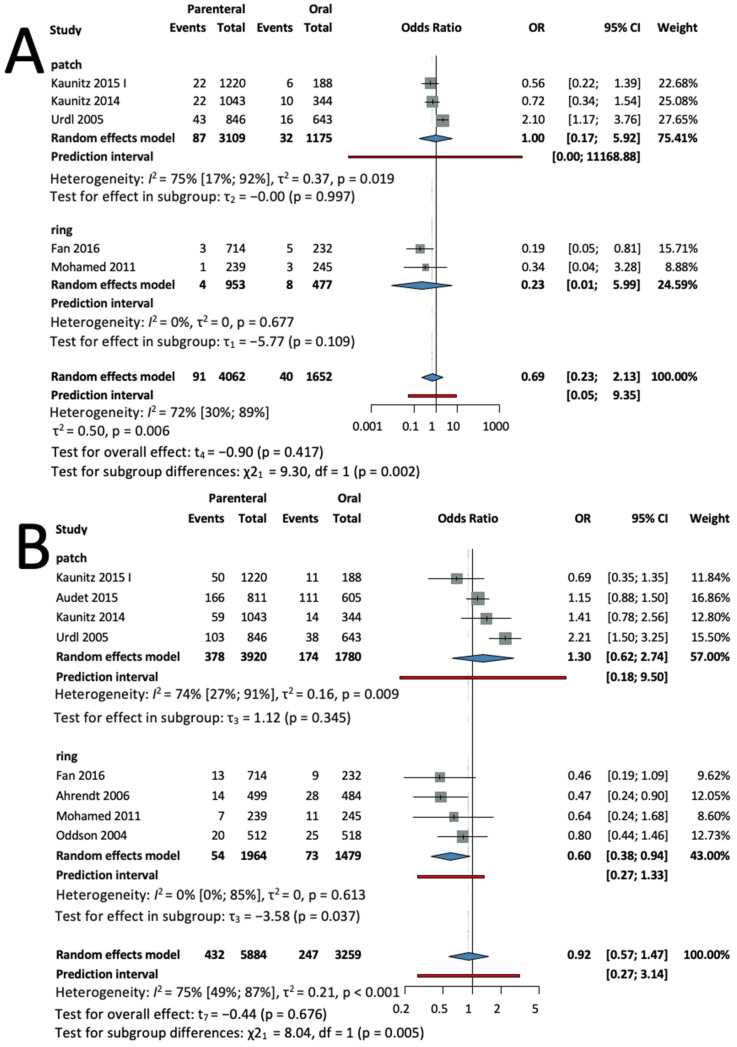
Forest plot of vomiting and nausea. (**A**) Forest plot of vomiting, comparing combined parenteral and oral hormonal contraceptives (**B**) Forest plot of nausea, comparing combined parenteral and oral hormonal contraceptives [28,30,32,33,35,37,38,39].

Five studies [28,32,33,35,39], with a total of 5715 patients, were selected for analysis. Decreased odds for vomiting tended to be found in the parenteral group, but this was not statistically significant. (OR = 0.71, CI: 0.23–2.14; I^2^ = 72%) (see Figure 5A).

In a subgroup analysis according to the method of administration, three studies [28,32,33], with a total of 4284 patients in the patch subgroup, found no difference compared to the oral group (*p* = 0.997, OR = 1.00, CI: 0.17–5.92; I^2^ = 76%) (see Figure 5A).

Two studies [35,39], with a total of 1430 patients in the ring subgroup, found reduced odds compared to the oral group. (OR = 0.23, CI: 0.01–5.99; I^2^ = 0%) (see Figure 5A).

The test for subgroup differences suggests that there was a statistically significant subgroup difference (χ^2^ = 9.30, df = 1, *p* = 0.002).

##### Nausea

Eight studies [28,30,32,33,35,37,38,39], with a total of 9143 patients, were selected for analysis. No difference was found for developing nausea in the parenteral group, but this was not statistically significant (OR = 0.96, CI: 0.61–1.52; I^2^ = 73%) (see Figure 5B).

In a subgroup analysis according to the method of administration, four studies [28,32,33,37], with a total of 5700 patients in the patch subgroup, found increased odds compared to the oral group (*p* =0.345, OR = 1.3, CI: 0.62–2.74; I^2^ = 65%) (see Figure 5B).

Four studies [30,35,38,39] in the ring subgroup, with a total of 3443 patients, found significantly reduced odds compared to the oral group (OR = 0.6, CI: 0.38–0.94; I^2^ = 0%) (see Figure 5B).

The test for subgroup differences suggests that there was a statistically significant subgroup difference (χ^2^ = 8.04, df = 1, *p* = 0.005).

#### 3.4.3. Vaginal Discharge

**Figure 6:**
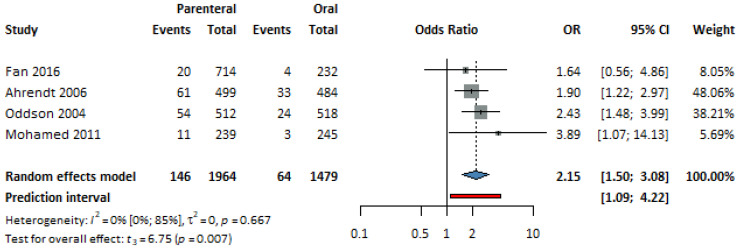
Forest plot of vaginal discharge, comparing combined parenteral and oral hormonal contraceptives [30,35,38,39].

Four studies [30,35,38,39], with a total of 3443 patients, were selected for analysis. The parenteral group had increased odds of vaginal discharge, and this was statistically significant (*p* = 0,007, OR = 2.15, CI: 1.5–3.08; I^2^ = 0%) (see Figure 6).

#### 3.4.4. Dysmenorrhea

**Figure 7:**
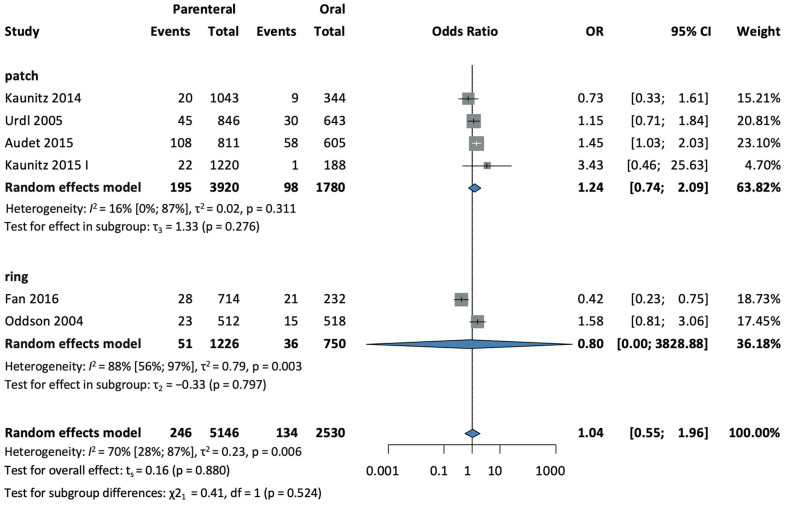
Forest plot of dysmenorrhea, comparing combined parenteral and oral hormonal contraceptives [28,30,32,33,35,37].

Six studies [28,30,32,33,35,37], with a total of 7676 patients, were selected for analysis. No statistical significance was found in the development of dysmenorrhea in the parenteral group. (OR = 0.94, CI: 0.55–1.62; I^2^ = 70% (95%) (see Figure 7).

In a subgroup analysis according to the method of administration, four studies [28,32,33,37], with a total of 5700 patients in the patch subgroup (OR = 1.24, CI: 0.74–2.09; I^2^ = 16%), and two studies [30,35], with a total of 1976 patients in the ring subgroup (OR = 0.8, CI: 0.0–3828; I^2^ = 88%), found no significant difference compared to oral group (see Figure 7).

The test for subgroup differences suggests that there was no statistically significant subgroup difference (χ^2^ = 0.41, df = 1, *p* = 0.524).

#### 3.4.5. Headache

**Figure 8:**
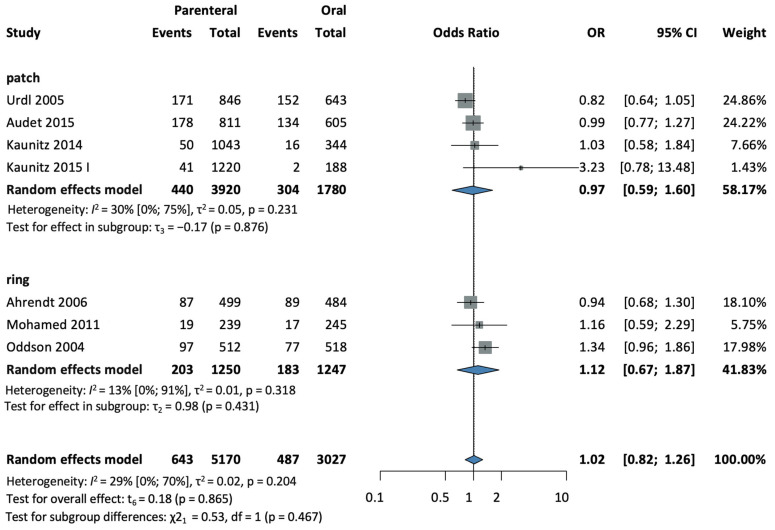
Forest plot of headache, comparing combined parenteral and oral hormonal contraceptives [28,30,32,33,37,38,39].

Seven studies [28,30,32,33,37,38,39], with a total of 8197 patients, were selected for analysis. There was no difference in the development of headaches between the parenteral and the oral administration groups. (OR = 0.97, CI: 0.81–1.16; I^2^ = 14%) (see Figure 8).

In a subgroup analysis according to the method of administration, four studies [28,32,33,37], with a total of 5700 patients in the patch subgroup, found slightly decreased odds compared to the oral group (OR = 0.97, CI: 0.59–1.60; I^2^ = 30%) (see Figure 8).

Three studies [30,38,39], with a total of 2497 patients in the ring subgroup, found slightly increased odds compared to the control (OR = 1.12, CI: 0.67–1.87; I^2^ = 13%) (see Figure 8).

The test for subgroup differences suggests that there was no statistically significant subgroup difference (χ^2^ = 0.53, df = 1, *p* = 0.467).

### 3.5. Risk of Bias Assessment

Most of the outcomes of the studies included in the meta-analysis were rated as having a low or moderate risk of bias. The risk of bias was low in thirty-nine, moderate in eight and high in three outcomes of studies included in the meta-analysis. The risk of bias assessment for all outcomes is shown in Supplementary Figures S1–S16.

### 3.6. Quality Assessment

Quality assessment scores for all outcomes are shown in Supplementary Table S3.

## 4. Discussion

This systematic review and meta-analysis evaluated the contraceptive effectiveness, compliance, and side effect profile of combined oral and combined parenteral methods of hormonal contraception.

Contraceptive compliance plays a crucial role in the safety and effectiveness of the method, which is largely influenced by the method of administration [40]. Compliance with a contraceptive method is highly dependent on its side effect profile, which is a constant challenge for pharmaceutical manufacturers [1].

Previous studies have shown that combined parenteral and oral contraceptives did not differ in efficacy [28,30,32,35,37,38,41]. Our results are consistent with the Cochrane Library’s 2013 systematic review. The three combined hormonal treatments (pill, patch, and ring) have similar contraceptive efficacy, according to the review’s authors’ conclusions [42]. Additionally, a 2017 meta-analysis comparing the effectiveness of combined oral contraception and vaginal ring found no differences in contraceptive efficacy [13].

In summary, our results confirmed the findings of previous studies, as we found similar contraceptive effectiveness for the combined parenteral contraceptive methods observed (transdermal patch, vaginal ring) compared to COCs. Effectiveness was also similar after a subgroup analysis of vaginal ring and transdermal patch. In addition, our results highlight some differences in the adverse event profiles of different methods.

As the effectiveness of a contraceptive method depends on compliance with the regimen, it is a crucial outcome to consider when assessing the efficacy of a contraceptive method [3,43]. Compliance was better with the parenteral methods, showing a difference in favor of the transdermal patch versus the vaginal ring in subgroup analysis. Patch users showed statistically significantly better compliance than COC users in four trials [25,28,32,37]. One trial showed no difference [31]. Compliance varied in four studies on the vaginal ring. Two found better compliance among ring users [34,38], one study found poorer compliance [35], and one showed no difference [30] between vaginal ring users and COC users.

In four trials, ring users had significantly more vaginal discharge than COC users [30,35,38,39]. Some authors investigated the effect of contraception on vaginal flora [44,45] and vaginal ring use [46]. They found an increased risk for leukorrhea but also an increased number of Lactobacilli in the vaginal flora, suggesting that it might have a protective role in vaginal dysbacteriosis; however, elevated discharge could pose a significant challenge for women, leading them to explore alternative administration methods for greater satisfaction.

Our results found higher odds of experiencing breast discomfort in the parenteral group, and we also found a significant difference between the ring and patch subgroups after performing a subgroup analysis. We found a statistically significant increase in the patch subgroup, as suggested by previous authors, indicating that the vaginal contraceptive ring might be more suitable for women with breast discomfort [47].

Regarding nausea, we found no difference between parenteral and oral administration but found a statistically significant difference between the patch and ring subgroups, indicating that ring users had less nausea during use, suggesting that clinicians may consider recommending this method to patients who are concerned about nausea. We found a lower risk of vomiting with parenteral methods. After performing a subgroup analysis, we found a statistically significant difference between the patch and ring subgroups, with the ring subgroup having a lower risk for vomiting. Altogether, parenteral methods are associated with a lower chance of vomiting and nausea. Choosing the right method might contribute to preventing early discontinuation.

Ring users generally had less nausea, breast discomfort, vomiting, and dysmenorrhea than patch users.

We found no significant difference in headache and dysmenorrhea; all methods were safe to use.

These results suggest that both methods of drug administration are highly effective. The selection of the most suitable contraceptive approach should be customized to accommodate the unique preferences and particular requirements of each patient.

### 4.1. Strengths and Limitations

Our study has several strengths that enhance its credibility. We included only randomized controlled studies, which provide the highest available evidence. Furthermore, it is worth highlighting that there had not been any previous meta-analyses on this particular topic in earlier years, emphasizing the originality and importance of our study in the academic realm.

In addition, the use of random effects models in our meta-analysis is a notable strength, as they account for heterogeneity among the included studies, providing a more conservative estimate of the overall treatment effect.

While our study brings forth significant findings, it is essential to acknowledge certain limitations that warrant consideration. Foremost among these limitations is the notable heterogeneity observed in the assessment of adverse events across the included trials. Additionally, the lack of subgrouping within our analysis represents another limitation, as it may have obscured potential variations in treatment effects across specific populations or intervention characteristics. Furthermore, a subset of these trials lacked comprehensive data concerning efficacy, adding a layer of complexity to the comprehensive analysis. Grey literature, which includes unpublished studies and reports, may not undergo the same scrutiny as peer-reviewed publications and can introduce potential biases. By focusing solely on RCTs published in peer-reviewed journals, we aimed to maintain a high level of methodological quality and enhance the validity of our meta-analysis.

We acknowledge that this exclusion is a limitation, and future research may consider incorporating grey literature to provide a more comprehensive overview of the available evidence in this field. Moreover, the varying utilization of the Pearl Index among studies poses a challenge, impeding direct comparisons between them and thereby influencing the overall coherence of the findings. These limitations underscore the need for caution when interpreting and generalizing the conclusions drawn from our study.

### 4.2. Implications for Research

Future research should delve deeper into the underlying factors influencing compliance and adverse events associated with different routes of hormonal contraceptive administration. Investigating patient-specific variables such as age, lifestyle, or hormonal sensitivities could offer valuable insights into why certain individuals might respond differently to specific administration methods. Longitudinal studies tracking adherence and side effects over extended periods could also provide a more comprehensive understanding of the sustained effects and variability among contraceptive users. Additionally, comparative studies exploring newer formulations or delivery systems within both oral and parenteral methods may further refine our understanding and potentially offer improved options for contraception.

It would be advisable to conduct randomized controlled trials, use a strict methodology in terms of reporting compliance, and collect data on adverse events. Introducing new drugs, such as progesterone-only patches and rings, is also a new option for research.

### 4.3. Implications for Practice

The application of scientific results to clinical practice is one of the biggest challenges in medicine today [48,49]. An accurate understanding of the side effect profile of oral as well as parenteral methods allows for a patient-centered, personalized therapeutic approach.

In accordance with the principles of translational medicine, our study was crafted with a commitment to fostering a connection between scientific inquiry and its implementation at the patient’s bedside.

## 5. Conclusions

Parenteral combined hormonal contraceptive methods demonstrate a commendable safety profile, exhibiting superior compliance compared to oral administration while maintaining comparable effectiveness. Our comprehensive findings underscore the distinct side effect profiles associated with these diverse modes of administration. This nuanced understanding can serve as a pivotal guide for clinicians, enabling them to discern and recommend a contraceptive modality that not only exhibits high effectiveness but also manifests a tailored, minimized side effect profile. This strategic selection is poised to yield enhanced compliance rates and reduced discontinuation instances and ultimately foster heightened patient satisfaction with contraceptive choices.

## Figures and Tables

**Table 1:** Characteristics of studies included.

First Author, Year of Publication	Methods, Country	Participants	Interventions	Outcomes	Total Patients in Parenteral	Total Patients in COC	Mean (SD) Age in Parenteral	Mean (SD) Age in COC
Ahrendt, 2006 [38]	Randomized trial in 10 European countries from May 2002 to Apr 2004.	1017 women, at least 18 years old.	Vaginal ring (etonogestrel 120 μg + EE 15 μg) versus COC (drospirenone 3 mg + EE 30 μg).	Contraceptive efficacy, compliance, acceptability, adverse events, continuation.	499	484	26.6 (6.1)	26.6 (6.2)
Audet, 2001 [37]	39 centers in the United States and 6 centers in Canada.	Healthy women aged 18 to 45 years.	Patch (norelgestromin 150 μg + EE 20 μg) versus oral contraceptive (levonorgestrel 50/75/125 μg + EE 30/40/30 μg).	Pearl Index, cycle control, compliance, adverse events.	811	605	28.0 (6.6)	27.8 (6.4)
Creinin, 2008 [36]		581 women.	Vaginal ring (etonogestrel 120 μg + EE 15 μg) versus transdermal patch (norelgestromin 150 μg + EE 20 μg).	Adverse events.	241	238	26.2 (5.6)	25.1 (5.5)
Fan, 2016 [35]	Phase-III, open-label, randomized multicenter trial in China.	1137 healthy women.	NuvaRing or COC.	Contraceptive efficacy, cycle control, dysmenorrhea, compliance.	714	232	31.8 (4.0)	31.2 (3.9)
Gilliam, 2010 [34]		273 women, 18–45 years.	Vaginal ring (etonogestrel 120 μg + EE 15 μg) versus COC.	Compliance.	135	137	22.3 (N/A)	22 (N/A)
Kaunitz, 2014 [32]	Open-label, randomized, parallel-group, multicenter study.	Women, 17–40 years of age.	Transdermal patch (120 μg levonorgestrel + 30 μg EE) versus 100 μg LNG and 20 μg EE.	Contraceptive efficacy, compliance, tolerability.	1043	344	26.4 (5.7)	26.4 (5.7)
Kaunitz, 2015 [25]	Open-label, randomized, multicenter, parallel-group clinical trial.	Women, 17–40 years of age.	Transdermal patch (120 μg levonorgestrel + 30 μg EE) versus 100 μg LNG and 20 μg EE.	Adverse events.	998	330	26.4 (N/A)	26.4 (N/A)
Kaunitz, 2015 [33]	Multicenter crossover study, presumably conducted in USA, dates not specified.	Women, 17–40 years.	Transdermal patch, (levonorgestrel 120 μg + EE 30 μg) versus COC (levonorgestrel 150 μg + EE 30 μg).	Pregnancy (Pearl Index), breakthrough bleeding and spotting, noncompliance, patch wearability, and adverse events.	1450	188	26.4 (5.6)	26.7 (5.7)
Merz, 2015 [31]	Double-blind, double-dummy, randomized, controlled, parallel-group, multicenter trial conducted at 28 centers in the United States.	Healthy women, 18–45 years of age.	Transdermal patch (0.55 mg EE and 2.1 mg GSD) versus COC (0.02 mg EE and 0.1 mg levonorgestrel).	Bleeding pattern, cycle control, safety.	199	198	29.1 (7.3)	27.2 (6.8)
Mohamed, 2011 [39]	Randomized trial conducted in Cairo, Egypt between 1 May 2008 and 31 July 2010.	600 women between 17 and 42 years.	Vaginal ring (etonogestrel 120 μg + EE 15 μg) versus COC (drospirenone 3 mg + EE 30 μg).	Cycle control (via diary cards), withdrawal bleeding, breakthrough bleeding or spotting, adverse events.	300	300	29.7 (4.1)	30.9 (4.2)
Oddsson, 2004 [30]	Open-label, randomized, comparative, multi-center trial in Belgium, Brazil, Chile, Denmark, Finland, France, Germany, Italy, Norway, Spain, and Sweden	1090 healthy women.		Contraceptive efficacy, cycle control, dysmenorrhea, compliance.	512	518	27.0 (6.2)	27.2 (6.3)
Rad, 2005 [29]	48 healthy premenopausal women between 18 and 34 years old.		Vaginal ring (nestorone 150 μg + EE 15 μg) versus COC (levonorgestrel 150 μg + EE 30 μg).	Pregnancy and continuation. Study focused on hemostasis variables.	23	24	24 (N/A)	N/A (N/A)
Urdl, 2005 [28]	Open-label, randomized trial in 65 centers in Europe and South Africa.	1517 healthy women aged 18 to 45 years.	Transdermal patch (norelgestromin 150 μg + EE 20 μg) versus COC (desogestrel 150 μg + EE 20 μg)	Pregnancy, continuation, compliance, cycle control, satisfaction, adverse events.	846	643	28.8 (6.5)	28.3 (6.5)

## Data Availability

The datasets used in this study can be found in the full-text articles included in the systematic review and meta-analysis.

## Supplementary Materials

The following supporting information can be downloaded at: https://www.mdpi.com/article/10.3390/jcm13020575/s1, Table S1: PRISMA 2020 checklist. Table S2: PRISMA 2020 for abstracts checklist. Table S3: Summary of Findings Table. Figure S1: Risk of bias assessment of the included studies assessing the Pearl index, using the revised tool for assessing risk of bias in randomized trials (Rob 2). Figure S2: Risk of bias assessment of the included studies assessing the Pearl index, broken down to tools, shown in percentage. Figure S3: Risk of bias assessment of the included studies assessing compliance, using the revised tool for assessing risk of bias in randomized trials (Rob 2). Figure S4: Risk of bias assessment of the included studies assessing compliance, broken down to tools, shown in percentage. Figure S5: Risk of bias assessment of the included studies assessing vomiting, using the revised tool for assessing risk of bias in randomized trials (Rob 2). Figure S6: Risk of bias assessment of the included studies assessing vomiting, broken down to tools, shown in percentage. Figure S7: Risk of bias assessment of the included studies assessing nausea, using the revised tool for assessing risk of bias in randomized trials (Rob 2). Figure S8: Risk of bias assessment of the included studies assessing nausea, broken down to tools, shown in percentage. Figure S9: Risk of bias assessment of the included studies assessing headache, using the revised tool for assessing risk of bias in randomized trials (Rob 2). Figure S10: Risk of bias assessment of the included studies assessing headache, broken down to tools, shown in percentage. Figure S11: Risk of bias assessment of the included studies assessing dysmenorrhea, using the revised tool for assessing risk of bias in randomized trials (Rob 2). Figure S12: Risk of bias assessment of the included studies assessing dysmenorrhea, broken down to tools, shown in percentage. Figure S13: Risk of bias assessment of the included studies assessing discharge, using the revised tool for assessing risk of bias in randomized trials (Rob 2). Figure S14: Risk of bias assessment of the included studies assessing discharge, broken down to tools, shown in percentage. Figure S15: Risk of bias assessment of the included studies assessing breast discomfort, using the revised tool for assessing risk of bias in randomized trials (Rob 2). Figure S16: Risk of bias assessment of the included studies assessing breast discomfort, broken down to tools, shown in percentage. Figure S17: Funnel plot for studies assessing Pearl index. Figure S18: Funnel plot for studies assessing compliance. Figure S19: Funnel plot for studies assessing vomiting. Figure S20: Funnel plot for studies assessing nausea. Figure S21: Funnel plot for studies assessing headache. Figure S22: Funnel plot for studies assessing dysmenorrhea. Figure S23: Funnel plot for studies assessing discharge. Figure S24: Funnel plot for studies assessing breast discomfort. Refs. [15,25,26,28,30,31,32,34,35,37,38,39] cited in Supplementary Materials file.
