# Supplementary material for: Comparison of Combined Parenteral and Oral Hormonal Contraceptives: A Systematic Review and Meta-Analysis of Randomized Trials
Source: J Clin Med. 2024 Jan 19;13(2):575. doi: 10.3390/jcm13020575 (PMC10816843; doi:10.3390/jcm13020575)
Supplement: Supplementary file 1 [file jcm-13-00575-s001.zip › jcm-2775336-supplementary.pdf]

SUPPLEMENTARY MATERIAL

Title

Comparing combined parenteral and oral hormonal contraceptives: a systematic review and meta-analysis of randomized trials.

Authors

Gábor Vleskó [1, 2], Fanni A. Meznerics [2, 3], Péter Hegyi [2, 4, 5], Brigitta Teutsch [2, 4], Márkó Unicsovics [1, 2], Zoltán Sipos [4, 8], Péter Fehérvári [2, 6], Nándor Ács [1, 2], *°Szabolcs Várbíró [1, 7], *Márton Keszthelyi [1, 2]

Affiliations

[1] Department of Obstetrics and Gynecology, Semmelweis University, 1082 Budapest, Hungary

[2] Centre for Translational Medicine, Semmelweis University, 1085 Budapest, Hungary

[3] Department of Dermatology, Venereology and Dermatooncology, Faculty of Medicine, Semmelweis University, Budapest, Hungary

[4] Institute for Translational Medicine, Medical School, University of Pécs, 7621 Pécs, Hungary

[5] Institute of Pancreatic Diseases, Semmelweis University, 1085 Budapest, Hungary

[6] Department of Biostatistics, University of Veterinary Medicine, 1078 Budapest, Hungary

[7] Workgroup of Research Management, Doctoral School, Semmelweis University, 1085 Budapest, Hungary

[8] Institute of Bioanalysis, Medical School, University of Pécs, Pécs, Hungary

* These authors contributed equally to this work.

Supplementary Table S1: PRISMA 2020 checklist(1)

Supplementary Table S2: PRISMA 2020 for abstracts checklist(1)

Supplementary Table S3: Summary of Findings Table

Supplementary Figure S1: Risk of bias assessment of the included studies assessing the Pearl index, using the revised tool for assessing risk of bias in randomized trials (Rob 2) (2)

Supplementary Figure S2: Risk of bias assessment of the included studies assessing the Pearl index, broken down to tools, shown in percentage

Supplementary Figure S3: Risk of bias assessment of the included studies assessing compliance, using the revised tool for assessing risk of bias in randomized trials (Rob 2)

Supplementary Figure S4: Risk of bias assessment of the included studies assessing compliance, broken down to tools, shown in percentage

Supplementary Figure S5: Risk of bias assessment of the included studies assessing vomiting, using the revised tool for assessing risk of bias in randomized trials (Rob 2)

Supplementary Figure S6: Risk of bias assessment of the included studies assessing vomiting, broken down to tools, shown in percentage

Supplementary Figure S7: Risk of bias assessment of the included studies assessing nausea, using the revised tool for assessing risk of bias in randomized trials (Rob 2)

Supplementary Figure S8: Risk of bias assessment of the included studies assessing nausea, broken down to tools, shown in percentage

Supplementary Figure S9: Risk of bias assessment of the included studies assessing headache, using the revised tool for assessing risk of bias in randomized trials (Rob 2)

Supplementary Figure S10: Risk of bias assessment of the included studies assessing headache, broken down to tools, shown in percentage

Supplementary Figure S11: Risk of bias assessment of the included studies assessing dysmenorrhea, using the revised tool for assessing risk of bias in randomized trials (Rob 2)

Supplementary Figure S12: Risk of bias assessment of the included studies assessing dysmenorrhea, broken down to tools, shown in percentage

Supplementary Figure S13: Risk of bias assessment of the included studies assessing discharge, using the revised tool for assessing risk of bias in randomized trials (Rob 2)

Supplementary Figure S14: Risk of bias assessment of the included studies assessing discharge, broken down to tools, shown in percentage

Supplementary Figure S15: Risk of bias assessment of the included studies assessing breast discomfort, using the revised tool for assessing risk of bias in randomized trials (Rob 2)

Supplementary Figure S16: Risk of bias assessment of the included studies assessing breast discomfort, broken down to tools, shown in percentage

Supplementary Figure S17: Funnel plot for studies assessing Pearl index

Supplementary Figure S18: Funnel plot for studies assessing compliance

Supplementary Figure S19: Funnel plot for studies assessing vomiting

Supplementary Figure S20: Funnel plot for studies assessing nausea

Supplementary Figure S21: Funnel plot for studies assessing headache

Supplementary Figure S22: Funnel plot for studies assessing dysmenorrhea

Supplementary Figure S23: Funnel plot for studies assessing discharge

Supplementary Figure S24: Funnel plot for studies assessing breast discomfort

Supplementary References

Supplementary Figure S1: Risk of bias assessment of Pearl index using the RoB 2 tool (3, 4, 5, 6, 7, 8)

Author, year	Intervention	Comparison	Outcome	D1	D2	D3	D4	D5	Overall
Audet 2015	patch	COC	Pearl index	+	+	+	-	!	-
Urdl 2005	patch	COC	Pearl Index	+	+	+	-	!	-
Kaunitz 2014	patch	COC	Pearl Index	+	-	-	-	!	-
Fan 2016	ring	COC	Pearl Index	+	+	+	+	-	-
Ahrendt 2006	ring	COC	Pearl Index	+	+	+	+	-	-
Oddson 2004	ring	COC	Pearl Index	+	+	+	!	-	-

\+ Low risk
! Some concerns
\- High risk

D1 Randomisation process
D2 Deviations from the intended interventions
D3 Missing outcome data
D4 Measurement of the outcome
D5 Selection of the reported result

Supplementary Figure S2: Risk of bias assessment of the included studies assessing the Pearl index, broken down to tools, shown in percentage (3, 4, 5, 6, 7, 8)

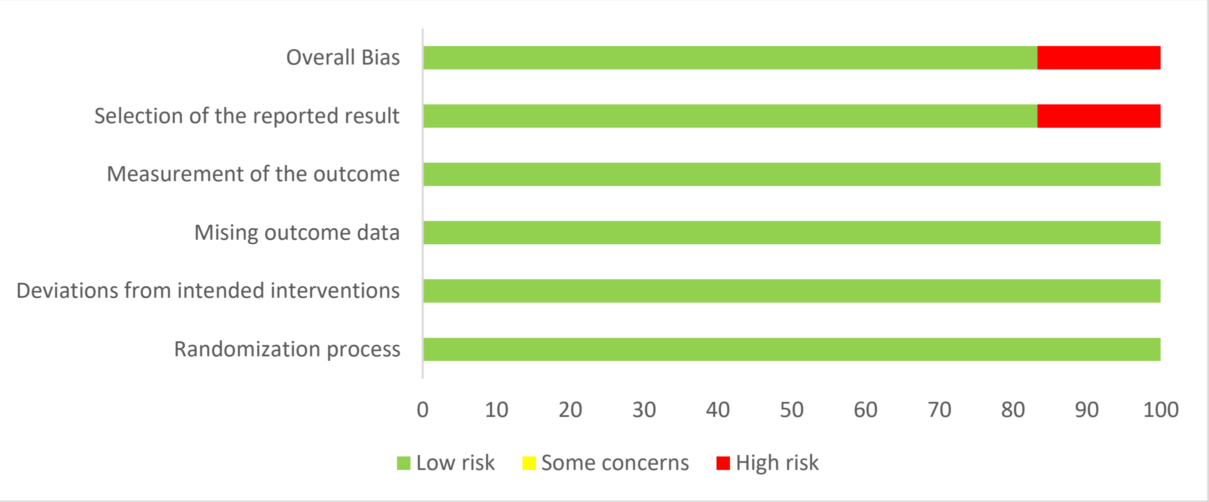

Supplementary Figure S3: Risk of bias assessment of compliance using the RoB 2 tool (3, 4, 5, 6, 7, 8, 9, 10, 11)

Author, year	Intervention	Comparison	Outcome	D1	D2	D3	D4	D5	Overall
Merz 2015	patch	COC	compliance	+	+	+	+	!	!
Audet 2015	patch	COC	compliance	+	+	+	-	!	-
Kaunitz 2014	patch	COC	compliance	+	-	-	-	!	-
Kaunitz 2015	patch	COC	compliance	+	-	-	-	!	-
Urdl 2005	patch	COC	compliance	+	+	+	-	!	-
Fan 2016	ring	COC	compliance	+	+	+	+	-	-
Oddson 2004	ring	COC	compliance	+	+	+	!	-	-
Ahrendt 2006	ring	COC	compliance	+	+	+	+	-	-
Gilliam 2010	ring	COC	compliance	+	+	+	-	!	-

\+ Low risk
! Some concerns
\- High risk

D1 Randomisation process
D2 Deviations from the intended interventions
D3 Missing outcome data
D4 Measurement of the outcome
D5 Selection of the reported result

Supplementary Figure S4: Risk of bias assessment of the included studies assessing compliance, broken down to tools, shown in percentage (3, 4, 5, 6, 7, 8, 9, 10, 11)

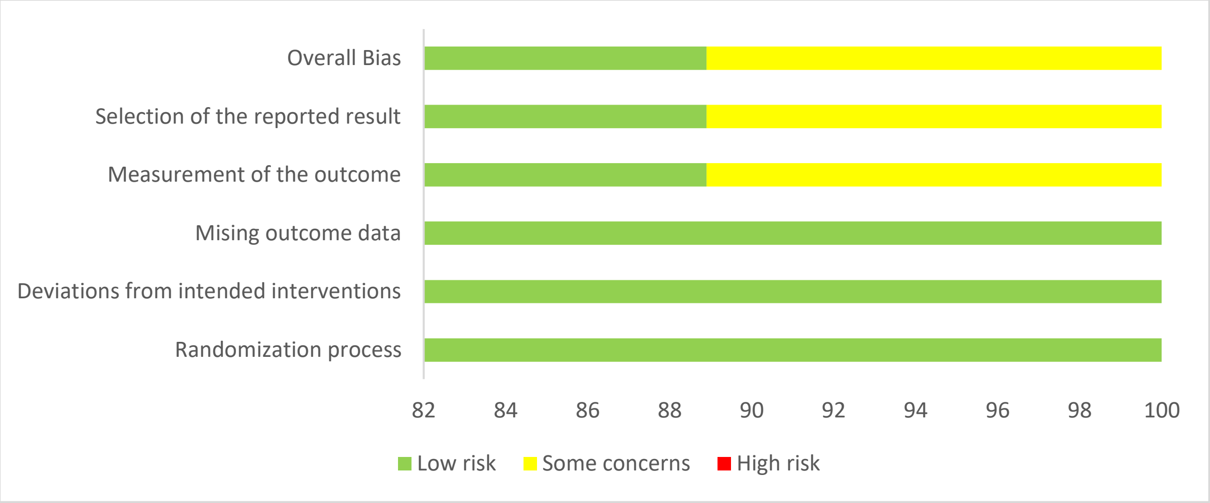

Supplementary Figure S5: Risk of bias assessment of vomiting using the RoB 2 tool (3, 5, 10)

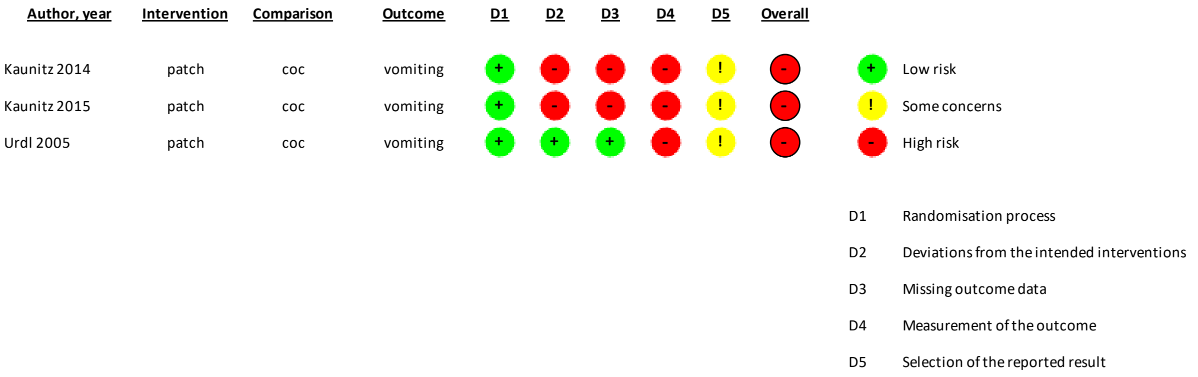

Supplementary Figure S6: Risk of bias assessment of the included studies assessing vomiting, broken down to tools, shown in percentage (3, 5, 10)

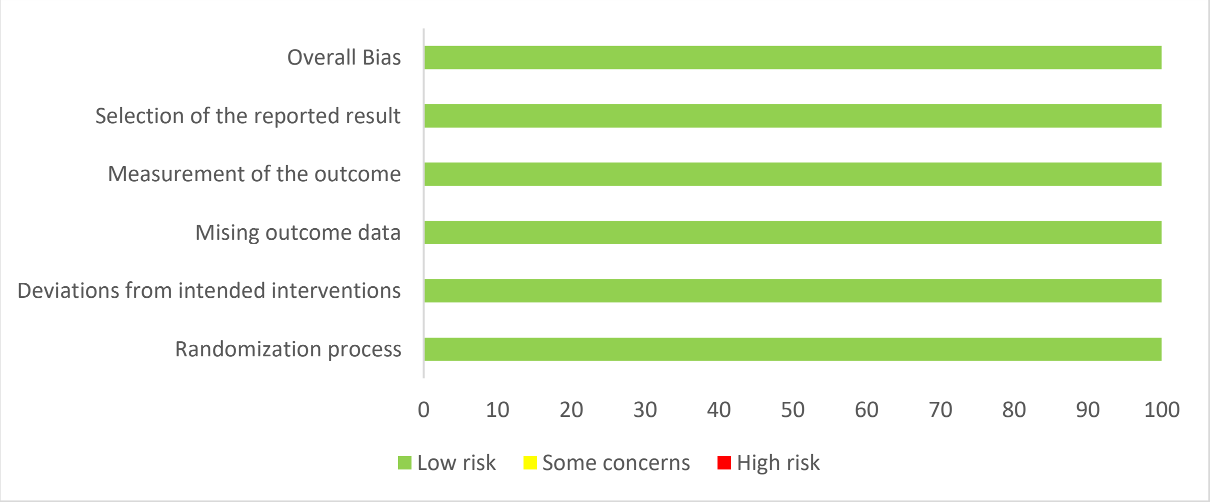

Supplementary Figure S7: Risk of bias assessment of nausea using the RoB 2 tool (3, 4, 5, 6, 7, 8, 10, 12)

Author, year	Intervention	Comparison	Outcome	D1	D2	D3	D4	D5	Overall
Kaunitz 2014	patch	COC	nausea	+	-	-	-	!	-
Audet 2015	patch	COC	nausea	+	+	+	-	!	-
Kaunitz 2015	patch	COC	nausea	+	-	-	-	!	-
Urdl 2005	patch	COC	nausea	+	+	+	-	!	-
Fan 2016	ring	COC	nausea	+	+	+	+	-	-
Ahrendt 2006	ring	COC	nausea	+	+	+	+	-	-
Mohamed 2011	ring	COC	nausea	+	+	+	!	!	!
Oddson 2004	ring	COC	nausea	+	+	+	!	-	-

+ Low risk
! Some concerns
- High risk

D1 Randomisation process
D2 Deviations from the intended interventions
D3 Missing outcome data
D4 Measurement of the outcome
D5 Selection of the reported result

Supplementary Figure S8: Risk of bias assessment of the included studies assessing nausea, broken down to tools, shown in percentage (3, 4, 5, 6, 7, 8, 10, 12)

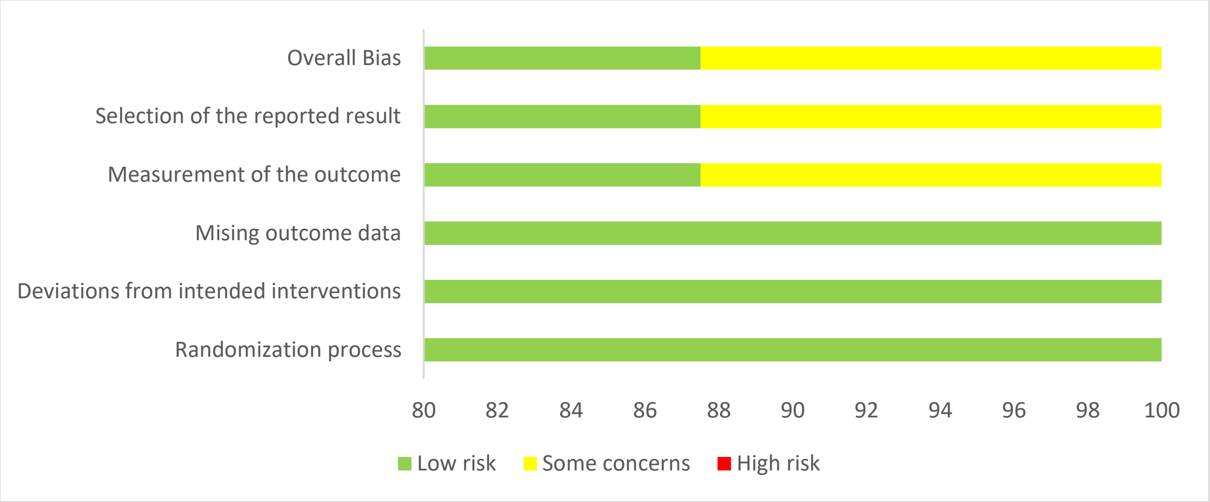

Supplementary Figure S9: Risk of bias assessment of headache using the RoB 2 tool (3, 4, 5, 7, 8, 10, 12)

Author, year	Intervention	Comparison	Outcome	D1	D2	D3	D4	D5	Overall
Kaunitz 2015	patch	COC	headache	+	-	-	-	!	-
Urdl 2005	patch	COC	headache	+	+	+	-	!	-
Audet 2015	patch	COC	headache	+	+	+	-	!	-
Kaunitz 2014	patch	COC	headache	+	-	-	-	!	-
Ahrendt 2006	ring	COC	headache	+	+	+	+	-	-
Mohamed 2011	ring	COC	headache	+	+	+	!	!	!
Oddson 2004	ring	COC	headache	+	+	+	!	-	-

+ Low risk
! Some concerns
- High risk

D1 Randomisation process
D2 Deviations from the intended interventions
D3 Missing outcome data
D4 Measurement of the outcome
D5 Selection of the reported result

Supplementary Figure S10: Risk of bias assessment of the included studies assessing headache, broken down to tools, shown in percentage (3, 4, 5, 7, 8, 10, 12)

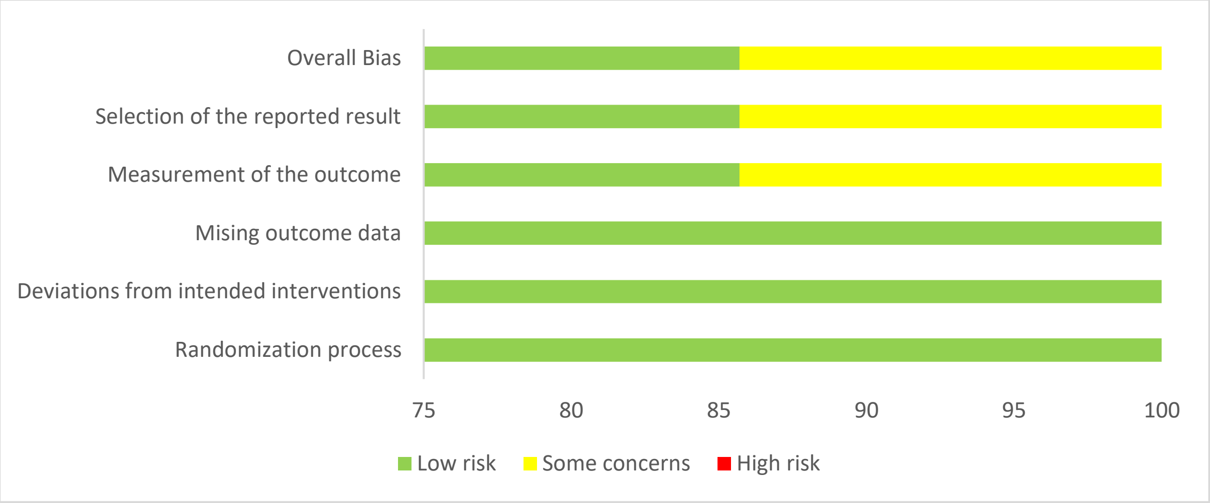

Supplementary Figure S11: Risk of bias assessment of dysmenorrhea using the RoB 2 tool (3, 4, 5, 6, 7, 10)

Author, year	Intervention	Comparison	Outcome	D1	D2	D3	D4	D5	Overall
Kaunitz 2015	patch	COC	dismenorrhoea	+	-	-	-	!	-
Kaunitz 2014	patch	COC	dismenorrhoea	+	-	-	-	!	-
Urdl 2005	patch	COC	dismenorrhoea	+	+	+	-	!	-
Audet 2015	patch	COC	dismenorrhoea	+	+	+	-	!	-
Fan 2016	ring	COC	dismenorrhoea	+	+	+	+	-	-
Oddson 2004	ring	COC	dismenorrhoea	+	+	+	!	-	-

+ Low risk
! Some concerns
- High risk

D1 Randomisation process
D2 Deviations from the intended interventions
D3 Missing outcome data
D4 Measurement of the outcome
D5 Selection of the reported result

Supplementary Figure S12: Risk of bias assessment of the included studies assessing dysmenorrhea, broken down to tools, shown in percentage

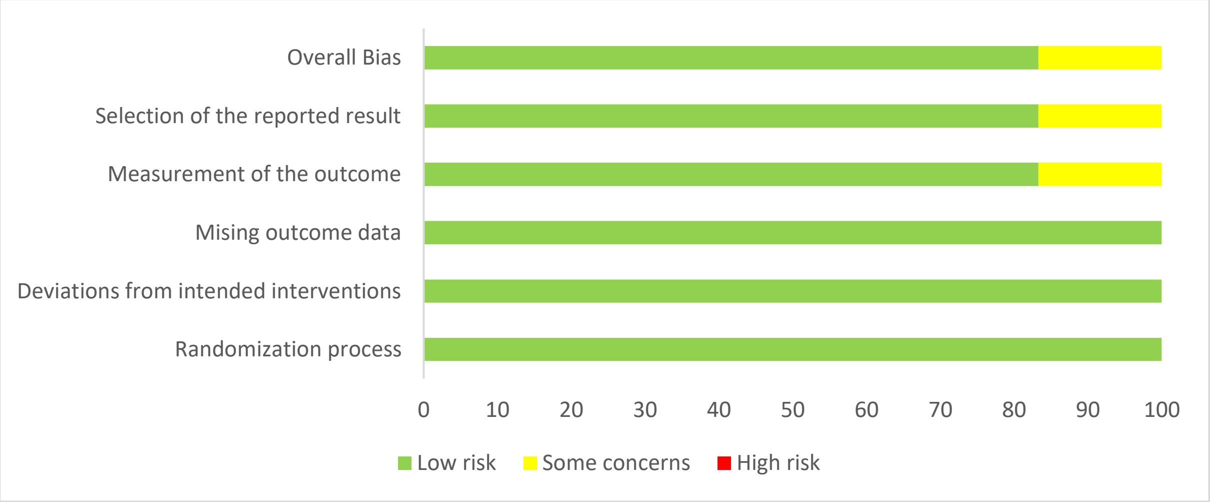

Supplementary Figure S13: Risk of bias assessment of discharge using the RoB 2 tool (4, 6, 8, 12)

Author, year	Intervention	Comparison	Outcome	D1	D2	D3	D4	D5	Overall
Fan 2016	ring	COC	discharge	+	+	+	+	-	-
Ahrendt 2006	ring	COC	discharge	+	+	+	+	-	-
Oddson 2004	ring	COC	discharge	+	+	+	!	-	-
Mohamed 2011	ring	COC	discharge	+	+	+	!	!	!

+ Low risk
! Some concerns
- High risk

D1 Randomisation process
D2 Deviations from the intended interventions
D3 Missing outcome data
D4 Measurement of the outcome
D5 Selection of the reported result

Supplementary Figure S14: Risk of bias assessment of the included studies assessing discharge, broken down to tools, shown in percentage

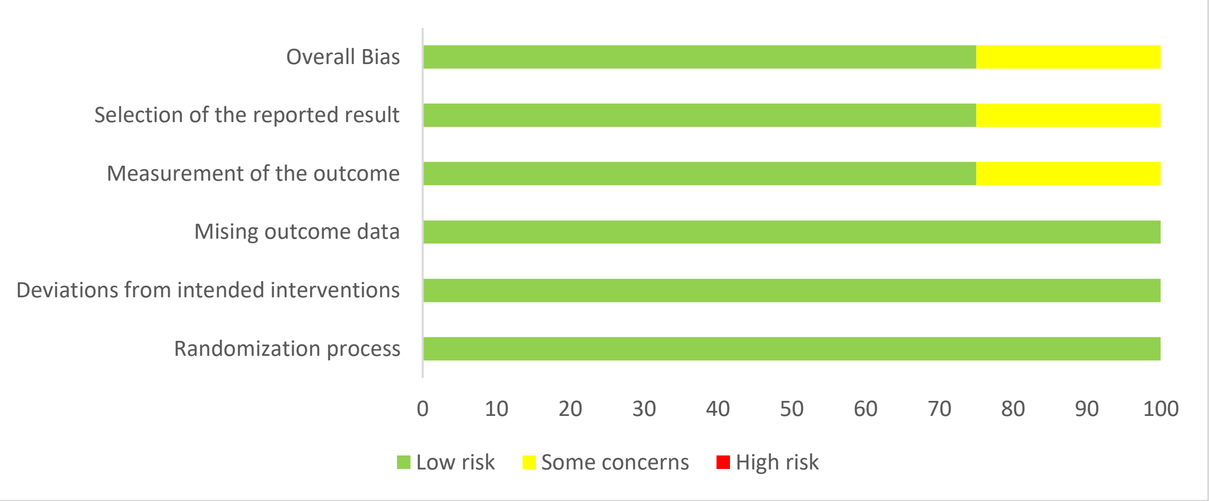

Supplementary Figure S15: Risk of bias assessment of breast discomfort using the RoB 2 tool (3, 4, 6, 7, 8, 10)

Author, year	Intervention	Comparison	Outcome	D1	D2	D3	D4	D5	Overall
Kaunitz 2015	patch	COC	breast discomfort	+	-	-	-	!	-
Kaunitz 2014	patch	COC	breast discomfort	+	-	-	-	!	-
Urdl 2005	patch	COC	breast discomfort	+	+	+	-	!	-
Audet 2015	patch	COC	breast discomfort	+	+	+	-	!	-
Ahrendt 2006	ring	COC	breast discomfort	+	+	+	+	-	-
Fan 2016	ring	COC	breast discomfort	+	+	+	+	-	-
Mohamed 2011	ring	COC	breast discomfort	+	+	+	!	!	!
Oddson 2004	ring	COC	breast discomfort	+	+	+	!	-	-

+ Low risk
! Some concerns
- High risk

D1 Randomisation process
D2 Deviations from the intended interventions
D3 Missing outcome data
D4 Measurement of the outcome
D5 Selection of the reported result

Supplementary Figure S16: Risk of bias assessment of the included studies assessing breast discomfort, broken down to tools, shown in percentage (3, 4, 6, 7, 8, 10)

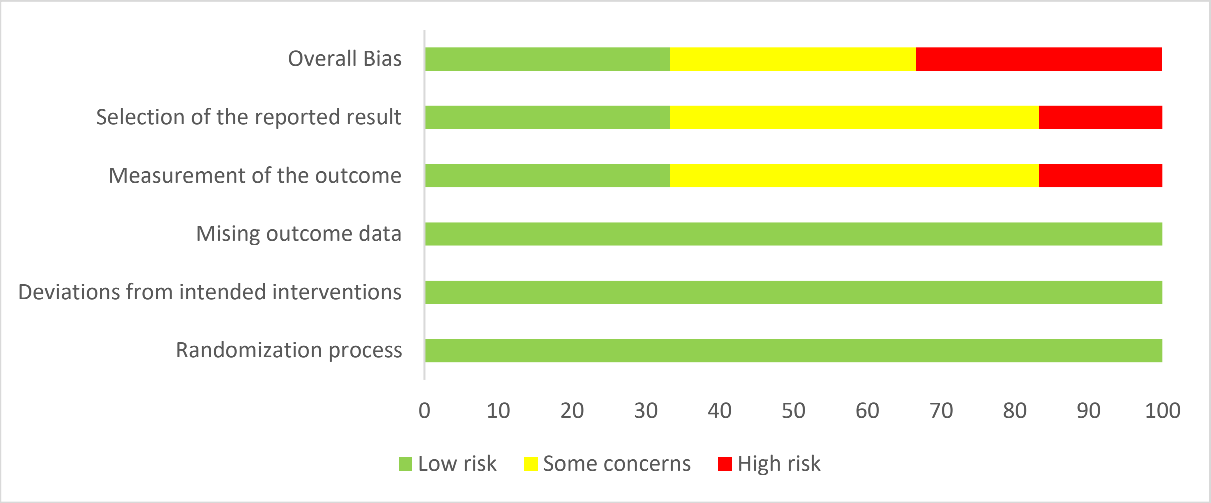

Supplementary Figure S17: Funnel plot showing publication bias of outcome Pearl index (3, 4, 5, 6, 7, 8)

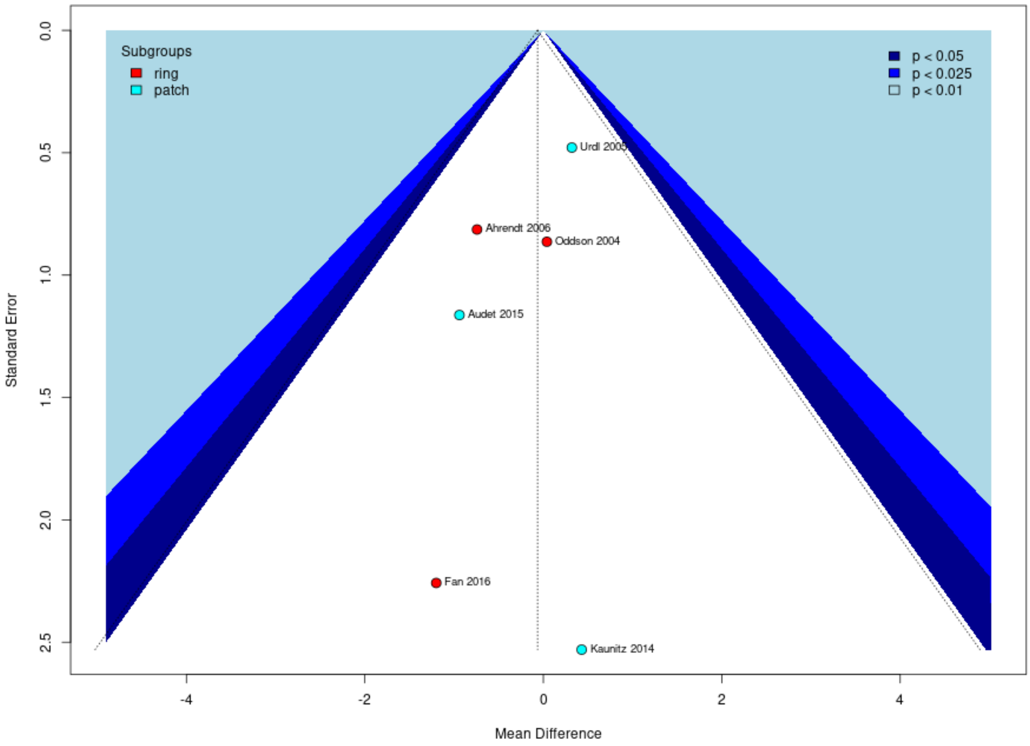

Supplementary Figure S18: Funnel plot showing publication bias of outcome compliance (3, 4, 5, 6, 7, 8, 9, 10, 11)

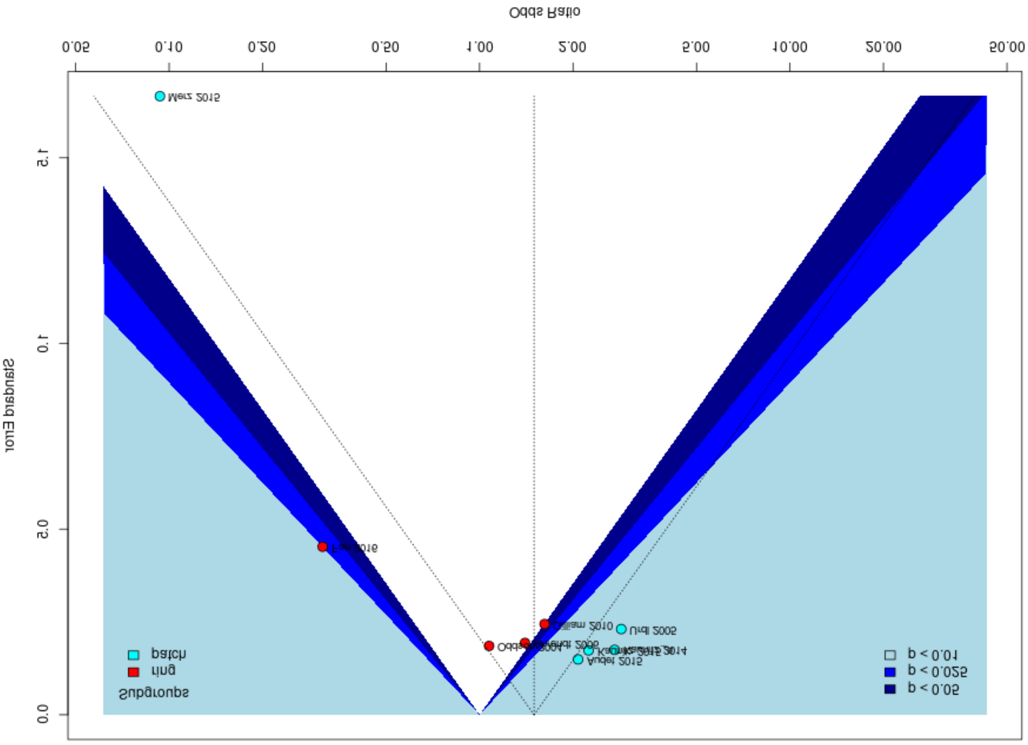

Supplementary Figure S19: Funnel plot showing publication bias of outcome vomiting (3, 5, 10)

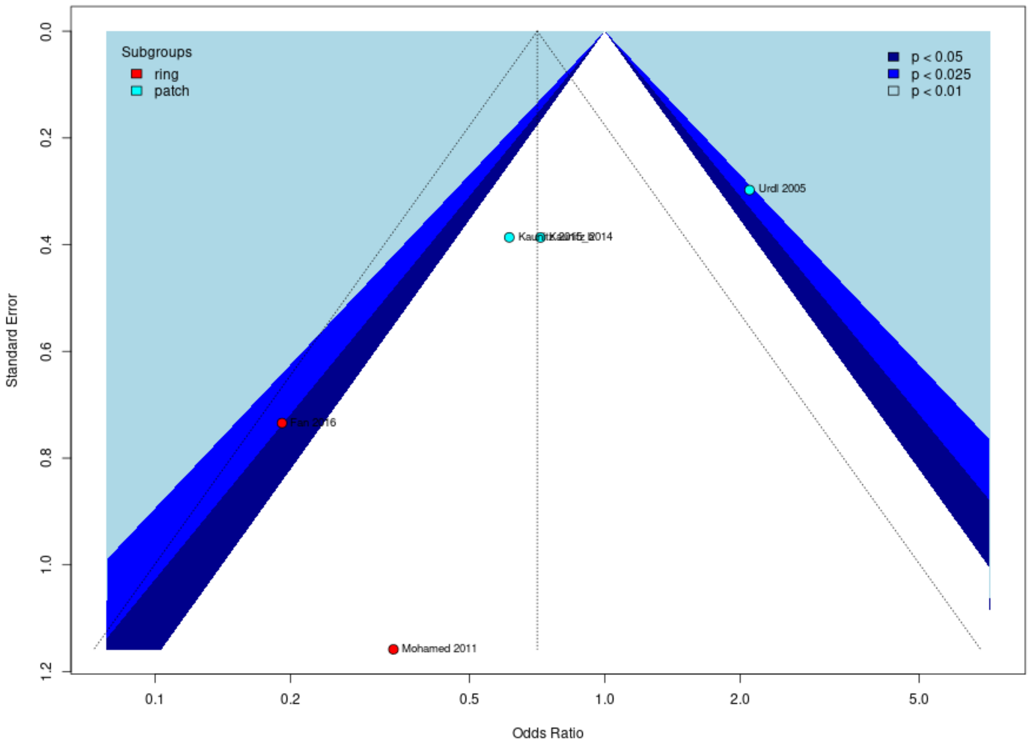

Supplementary Figure S20: Funnel plot showing publication bias of outcome nausea (3, 4, 5, 6, 7, 8, 10, 12)

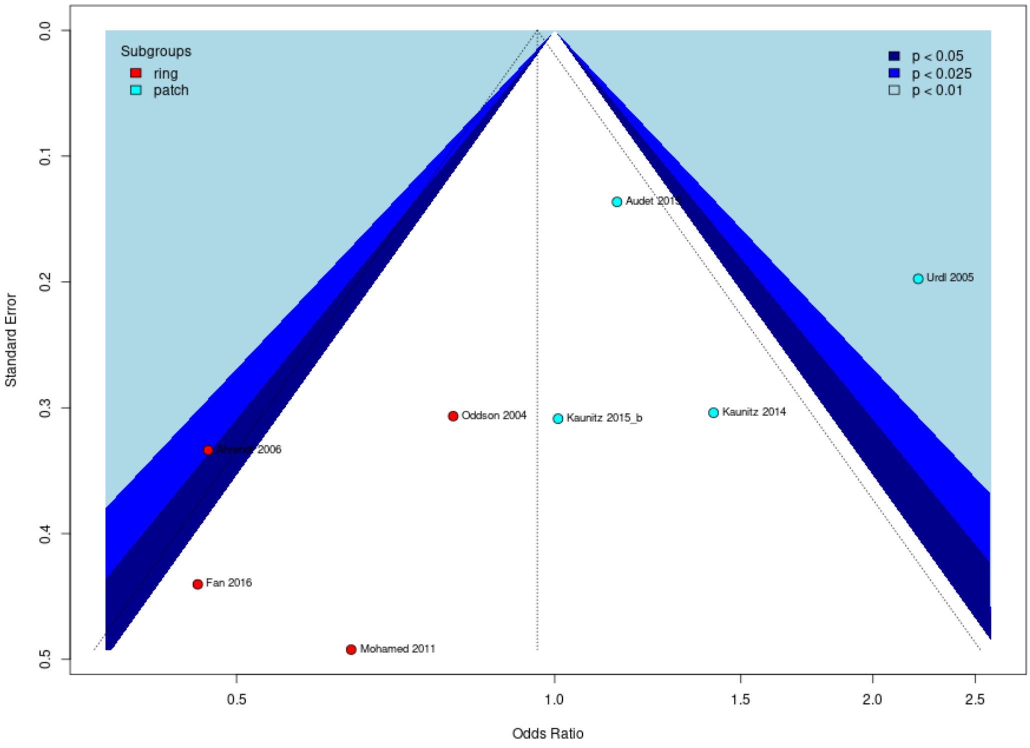

Supplementary Figure S21: Funnel plot showing publication bias of outcome headache (3, 4, 5, 7, 8, 10, 12)

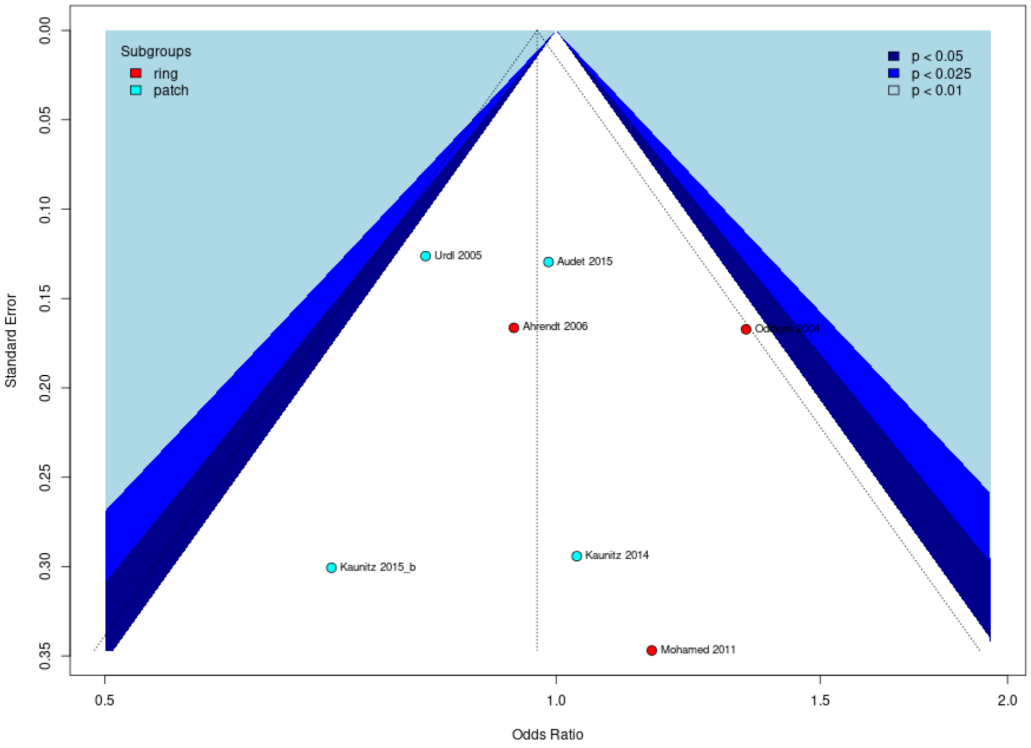

Supplementary Figure S22: Funnel plot showing publication bias of outcome dysmenorrhea (3, 4, 5, 6, 7, 10)

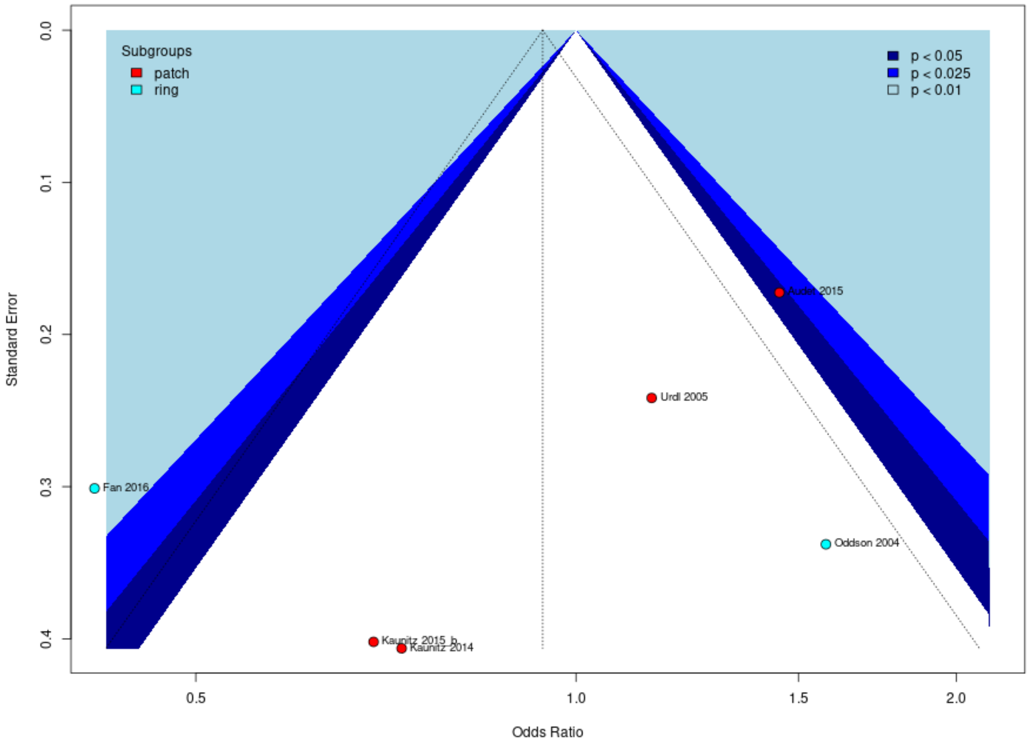

Supplementary Figure S23: Funnel plot showing publication bias of outcome discharge (4, 6, 8, 12)

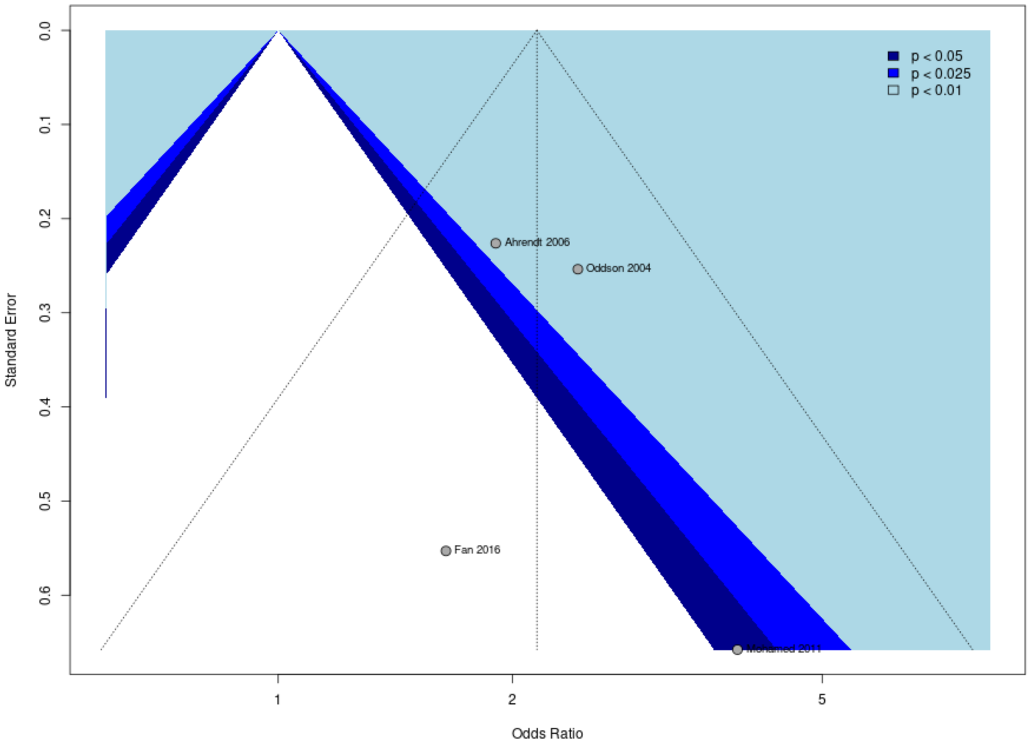

Supplementary Figure S24: Funnel plot showing publication bias of outcome breast discomfort (3, 4, 6, 7, 8, 10)

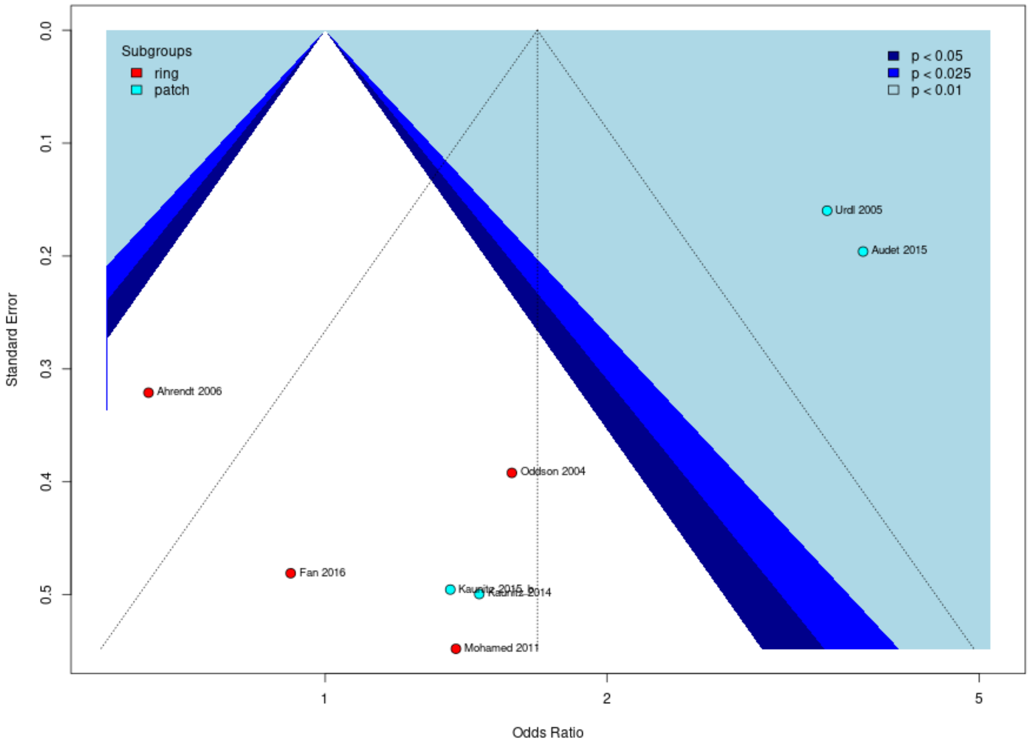

Supplementary Table S1: PRISMA 2020 checklist (1)

Section and Topic	Item #	Checklist item	Location where item is reported
**TITLE**			
Title	1	Identify the report as a systematic review.	1
**ABSTRACT**			
Abstract	2	See the PRISMA 2020 for Abstracts checklist.	Supp.T.S2
**INTRODUCTION**			
Rationale	3	Describe the rationale for the review in the context of existing knowledge.	4
Objectives	4	Provide an explicit statement of the objective(s) or question(s) the review addresses.	4
**METHODS**			
Eligibility criteria	5	Specify the inclusion and exclusion criteria for the review and how studies were grouped for the syntheses.	5-6
Information sources	6	Specify all databases, registers, websites, organisations, reference lists and other sources searched or consulted to identify studies. Specify the date when each source was last searched or consulted.	5
Search strategy	7	Present the full search strategies for all databases, registers and websites, including any filters and limits used.	5
Selection process	8	Specify the methods used to decide whether a study met the inclusion criteria of the review, including how many reviewers screened each record and each report retrieved, whether they worked independently, and if applicable, details of automation tools used in the process.	5-6
Data collection process	9	Specify the methods used to collect data from reports, including how many reviewers collected data from each report, whether they worked independently, any processes for obtaining or confirming data from study investigators, and if applicable, details of automation tools used in the process.	5-6
Data items	10a	List and define all outcomes for which data were sought. Specify whether all results that were compatible with each outcome domain in each study were sought (e.g. for all measures, time points, analyses), and if not, the methods used to decide which results to collect.	6
	10b	List and define all other variables for which data were sought (e.g. participant and intervention characteristics, funding sources). Describe any assumptions made about any missing or unclear information.	6
Study risk of bias assessment	11	Specify the methods used to assess risk of bias in the included studies, including details of the tool(s) used, how many reviewers assessed each study and whether they worked independently, and if applicable, details of automation tools used in the process.	6
Effect measures	12	Specify for each outcome the effect measure(s) (e.g. risk ratio, mean difference) used in the synthesis or presentation of results.	5-6
Synthesis methods	13a	Describe the processes used to decide which studies were eligible for each synthesis (e.g. tabulating the study intervention characteristics and comparing against the planned groups for each synthesis (item #5)).	5-6
	13b	Describe any methods required to prepare the data for presentation or synthesis, such as handling of missing summary statistics, or data conversions.	5-6
	13c	Describe any methods used to tabulate or visually display results of individual studies and syntheses.	6
	13d	Describe any methods used to synthesize results and provide a rationale for the choice(s). If meta-analysis was performed, describe the model(s), method(s) to identify the presence and extent of statistical heterogeneity, and software package(s) used.	5-6
	13e	Describe any methods used to explore possible causes of heterogeneity among study results (e.g. subgroup analysis, meta-regression).	5-6
	13f	Describe any sensitivity analyses conducted to assess robustness of the synthesized results.	5-6

Section and Topic	Item #	Checklist item	Location where item is reported
Reporting bias assessment	14	Describe any methods used to assess risk of bias due to missing results in a synthesis (arising from reporting biases).	6
Certainty assessment	15	Describe any methods used to assess certainty (or confidence) in the body of evidence for an outcome.	6
**RESULTS**			
Study selection	16a	Describe the results of the search and selection process, from the number of records identified in the search to the number of studies included in the review, ideally using a flow diagram.	7
	16b	Cite studies that might appear to meet the inclusion criteria, but which were excluded, and explain why they were excluded.	8
Study characteristics	17	Cite each included study and present its characteristics.	16
Risk of bias in studies	18	Present assessments of risk of bias for each included study.	S1-16
Results of individual studies	19	For all outcomes, present, for each study: (a) summary statistics for each group (where appropriate) and (b) an effect estimate and its precision (e.g. confidence/credible interval), ideally using structured tables or plots.	9-16
Results of syntheses	20a	For each synthesis, briefly summarise the characteristics and risk of bias among contributing studies.	16
	20b	Present results of all statistical syntheses conducted. If meta-analysis was done, present for each the summary estimate and its precision (e.g. confidence/credible interval) and measures of statistical heterogeneity. If comparing groups, describe the direction of the effect.	9-16
	20c	Present results of all investigations of possible causes of heterogeneity among study results.	6,16
	20d	Present results of all sensitivity analyses conducted to assess the robustness of the synthesized results.	16
Reporting biases	21	Present assessments of risk of bias due to missing results (arising from reporting biases) for each synthesis assessed.	16
Certainty of evidence	22	Present assessments of certainty (or confidence) in the body of evidence for each outcome assessed.	16
**DISCUSSION**			
Discussion	23a	Provide a general interpretation of the results in the context of other evidence.	17
	23b	Discuss any limitations of the evidence included in the review.	17-18
	23c	Discuss any limitations of the review processes used.	18
	23d	Discuss implications of the results for practice, policy, and future research.	19
**OTHER INFORMATION**			
Registration and protocol	24a	Provide registration information for the review, including register name and registration number, or state that the review was not registered.	5
	24b	Indicate where the review protocol can be accessed, or state that a protocol was not prepared.	5
	24c	Describe and explain any amendments to information provided at registration or in the protocol.	5
Support	25	Describe sources of financial or non-financial support for the review, and the role of the funders or sponsors in the review.	2
Competing interests	26	Declare any competing interests of review authors.	1
Availability of data, code and other materials	27	Report which of the following are publicly available and where they can be found: template data collection forms; data extracted from included studies; data used for all analyses; analytic code; any other materials used in the review.	5

Supplementary Table S2: PRISMA 2020 for abstracts checklist (1)

Section and Topic	Item #	Checklist item	Reported (Yes/No)
**TITLE**			
Title	1	Identify the report as a systematic review.	Yes
**BACKGROUND**			
Objectives	2	Provide an explicit statement of the main objective(s) or question(s) the review addresses.	Yes
**METHODS**			
Eligibility criteria	3	Specify the inclusion and exclusion criteria for the review.	Yes
Information sources	4	Specify the information sources (e.g. databases, registers) used to identify studies and the date when each was last searched.	Yes
Risk of bias	5	Specify the methods used to assess risk of bias in the included studies.	No
Synthesis of results	6	Specify the methods used to present and synthesise results.	No
**RESULTS**			
Included studies	7	Give the total number of included studies and participants and summarise relevant characteristics of studies.	No
Synthesis of results	8	Present results for main outcomes, preferably indicating the number of included studies and participants for each. If meta-analysis was done, report the summary estimate and confidence/credible interval. If comparing groups, indicate the direction of the effect (i.e. which group is favoured).	Yes
**DISCUSSION**			
Limitations of evidence	9	Provide a brief summary of the limitations of the evidence included in the review (e.g. study risk of bias, inconsistency and imprecision).	No
Interpretation	10	Provide a general interpretation of the results and important implications.	Yes
**OTHER**			
Funding	11	Specify the primary source of funding for the review.	No
Registration	12	Provide the register name and registration number.	No

Supplementary Table S3: Summary of Findings Table

Outcomes	Anticipated absolute effects* (95% CI)		Relative effect (95% CI)	№ of participants (studies)	Certainty of the evidence (GRADE)
	**Risk with Combined Oral Contraceptive**	**Risk with Combined Parenteral Hormonal Contraceptive**			
Pearl index PATCH (Efficacy) assessed with: pearl-index Scale from: 0 to 100	The mean pearl index PATCH was **0**	MD **0.25 higher** (1.19 lower to 1.48 higher)	-	4292 (3 RCTs)	⨁⨁⨁⨁ High
Pearl index RING	The mean pearl index RING was **0**	MD **0.43 lower** (1.73 lower to 0.88 higher)	-	2959 (3 RCTs)	⨁⨁⨁⨁ High
Compilance PATCH	848 per 1 000	**923 per 1 000** (845 to 963)	**OR 2.16** (0.98 to 4.72)	6017 (5 RCTs)	⨁⨁⨁◯ Moderate
Compilance RING	839 per 1 000	**841 per 1 000** (652 to 938)	**OR 1.02** (0.36 to 2.90)	3002 (4 RCTs)	⨁⨁⨁⨁ High
Headache PATCH	164 per 1 000	**180 per 1 000** (116 to 269)	**OR 1.12** (0.67 to 1.87)	5856 (4 RCTs)	⨁⨁⨁◯ Moderate
Headache RING	147 per 1 000	**162 per 1 000** (103 to 243)	**OR 1.12** (0.67 to 1.87)	2497 (3 RCTs)	⨁⨁⨁⨁ High
Discharge RING	43 per 1 000	**87 per 1 000** (62 to 120)	**OR 2.11** (1.47 to 3.01)	3443 (4 RCTs)	⨁⨁⨁⨁ High
Vomiting PATCH	27 per 1 000	**27 per 1 000** (5 to 130)	**OR 0.98** (0.18 to 5.39)	4440 (3 RCTs)	⨁⨁⨁⨁ High
Vomiting RING	17 per 1 000	**12 per 1 000** (4 to 35)	**OR 0.71** (0.23 to 2.14)	1430 (2 RCTs)	⨁⨁⨁⨁ High
Breast discomfort PATCH	53 per 1 000	**122 per 1 000** (55 to 251)	**OR 2.50** (1.04 to 6.03)	5856 (4 RCTs)	⨁⨁⨁⨁ High
Breast discomfort RING	32 per 1 000	**32 per 1 000** (16 to 61)	**OR 0.98** (0.49 to 1.95)	3443 (4 RCTs)	⨁⨁⨁⨁ High

Outcomes	Anticipated absolute effects* (95% CI)		Relative effect (95% CI)	№ of participants (studies)	Certainty of the evidence (GRADE)
	**Risk with Combined Oral Contraceptive**	**Risk with Combined Parenteral Hormonal Contraceptive**			
Dismenorrhoea PATCH	55 per 1 000	**58 per 1 000** (34 to 99)	**OR 1.07** (0.60 to 1.90)	5856 (4 RCTs)	⨁⨁⨁⨁ High
Dismenorrhoea RING	48 per 1 000	**39 per 1 000** (0 to 995)	**OR 0.80** (0.00 to 3640.15)	2016 (2 RCTs)	⨁⨁⨁⨁ High
Nausea PATCH	91 per 1 000	**122 per 1 000** (74 to 196)	**OR 1.38** (0.79 to 2.42)	5856 (4 RCTs)	⨁⨁⨁⨁ High
Nausea RING	49 per 1 000	**30 per 1 000** (19 to 47)	**OR 0.60** (0.38 to 0.95)	3443 (4 RCTs)	⨁⨁⨁⨁ High

Supplementary references

1. Page MJ, McKenzie JE, Bossuyt PM, Boutron I, Hoffmann TC, Mulrow CD, et al. The PRISMA 2020 statement: an updated guideline for reporting systematic reviews. Bmj. 2021;372:n71.
2. Sterne JAC, Savović J, Page MJ, Elbers RG, Blencowe NS, Boutron I, et al. RoB 2: a revised tool for assessing risk of bias in randomised trials. BMJ. 2019;366:l4898.
3. Urdl W, Apter D, Alperstein A, Koll P, Schönian S, Bringer J, et al. Contraceptive efficacy, compliance and beyond: factors related to satisfaction with once-weekly transdermal compared with oral contraception. Eur J Obstet Gynecol Reprod Biol. 2005;121(2):202-10.
4. Oddsson K, Leifels-Fischer B, de Melo NR, Wiel-Masson D, Benedetto C, Verhoeven CH, et al. Efficacy and safety of a contraceptive vaginal ring (NuvaRing) compared with a combined oral contraceptive: a 1-year randomized trial. Contraception. 2005;71(3):176-82.
5. Kaunitz AM, Portman D, Westhoff CL, Archer DF, Mishell DR, Jr., Rubin A, et al. Low-dose levonorgestrel and ethinyl estradiol patch and pill: a randomized controlled trial. Obstet Gynecol. 2014;123(2 Pt 1):295-303.
6. Fan GS, Ren M, Di W, Su P, Chang Q, Wu S, et al. Efficacy and safety of the contraceptive vaginal ring (NuvaRing) compared with a combined oral contraceptive in Chinese women: a 1-year randomised trial. European Journal of Contraception and Reproductive Health Care. 2016;21(4):303-9.
7. Audet MC, Moreau M, Koltun WD, Waldbaum AS, Shangold G, Fisher AC, et al. Evaluation of contraceptive efficacy and cycle control of a transdermal contraceptive patch vs an oral contraceptive: a randomized controlled trial. JAMA. 2001;285(18):2347-54.
8. Ahrendt HJ, Nisand I, Bastianelli C, Gómez MA, Gemzell-Danielsson K, Urdl W, et al. Efficacy, acceptability and tolerability of the combined contraceptive ring, NuvaRing, compared with an oral contraceptive containing 30 microg of ethinyl estradiol and 3 mg of drospirenone. Contraception. 2006;74(6):451-7.
9. Merz M, Kroll R, Lynen R, Bangerter K. Bleeding pattern and cycle control of a low-dose transdermal contraceptive patch compared with a combined oral contraceptive: a randomized study. Contraception. 2015;91(2):113-20.
10. Kaunitz AM, Portman D, Westhoff CL, Archer DF, Mishell DR, Foegh M. Self-reported and verified compliance in a phase 3 clinical trial of a novel low-dose contraceptive patch and pill. Contraception. 2015;91(3):204-10.
11. Gilliam ML, Neustadt A, Kozloski M, Mistretta S, Tilmon S, Godfrey E. Adherence and acceptability of the contraceptive ring compared with the pill among students: a randomized controlled trial. Obstetrics and gynecology. 2010;115(3):503-10.
12. Mohamed AMM, El-Sherbiny WSM, Mostafa WAI. Combined contraceptive ring versus combined oral contraceptive (30-μg ethinylestradiol and 3-mg drospirenone). International Journal of Gynecology and Obstetrics. 2011;114(2):145-8.
